# Co-crystal structures of HIV TAR RNA bound to lab-evolved proteins show key roles for arginine relevant to the design of cyclic peptide TAR inhibitors

**DOI:** 10.1074/jbc.RA120.015444

**Published:** 2021-01-13

**Authors:** Sai Shashank Chavali, Sachitanand M. Mali, Jermaine L. Jenkins, Rudi Fasan, Joseph E. Wedekind

**Affiliations:** 1 Department of Biochemistry and Biophysics and Center for RNA Biology, University of Rochester School of Medicine and Dentistry, Rochester, New York, USA; 2 Department of Chemistry, University of Rochester, Rochester, New York, USA

**Keywords:** HIV TAR, HIV Tat, RNA-protein interactions, cyclic peptide inhibitor, arginine-rich domain, drug discovery, isothermal titration calorimetry, surface plasmon resonance, peptide chemical synthesis, X-ray crystallography, human immunodeficiency virus (HIV), RNA-binding protein, RNA structure, RNA-protein interaction, cyclic peptide, surface plasmon resonance (SPR), isothermal titration calorimetry (ITC)

## Abstract

RNA-protein interfaces control key replication events during the HIV-1 life cycle. The viral *trans*-activator of transcription (Tat) protein uses an archetypal arginine-rich motif (ARM) to recruit the host positive transcription elongation factor b (pTEFb) complex onto the viral *trans*-activation response (TAR) RNA, leading to activation of HIV transcription. Efforts to block this interaction have stimulated production of biologics designed to disrupt this essential RNA-protein interface. Here, we present four co-crystal structures of lab-evolved TAR-binding proteins (TBPs) in complex with HIV-1 TAR. Our results reveal that high-affinity binding requires a distinct sequence and spacing of arginines within a specific β2-β3 hairpin loop that arose during selection. Although loops with as many as five arginines were analyzed, only three arginines could bind simultaneously with major-groove guanines. Amino acids that promote backbone interactions within the β2-β3 loop were also observed to be important for high-affinity interactions. Based on structural and affinity analyses, we designed two cyclic peptide mimics of the TAR-binding β2-β3 loop sequences present in two high-affinity TBPs (*K_D_* values of 4.2 ± 0.3 and 3.0 ± 0.3 nm). Our efforts yielded low-molecular weight compounds that bind TAR with low micromolar affinity (*K_D_* values ranging from 3.6 to 22 μm). Significantly, one cyclic compound within this series blocked binding of the Tat-ARM peptide to TAR in solution assays, whereas its linear counterpart did not. Overall, this work provides insight into protein-mediated TAR recognition and lays the ground for the development of cyclic peptide inhibitors of a vital HIV-1 RNA-protein interaction.

RNA-protein interactions play a central role in cellular processes that underlie health and human disease (1, 2, 3). As a case in point, the HIV life cycle requires a series of programmed exchanges whereby viral factors co-opt host functions to support proviral DNA transcription (4, 5). Early in this process, the viral protein Tat binds to the host positive transcription elongation factor b (pTEFb) complex, freeing it from an inactive state in which it is bound to host 7SK RNA (Fig. 1*A*). Tat utilizes a nine-amino acid arginine-rich motif (ARM) to supplant an ARM-like counterpart contributed by the host protein HEXIM. Upon its release, pTEFb is escorted by Tat to the viral *trans*-activation response (TAR) element (7, 10, 11, 12, 13, 14). TAR is an ∼59-nucleotide RNA located in the 5′-LTR of all HIV transcripts. TAR comprises an A-form helical stem loop punctuated by a central trinucleotide bulge and capped by an apical hexaloop. Collectively, these elements compose a structure that is essential for activity (10, 15, 16, 17, 18). The Tat ARM further recognizes TAR and delivers the pTEFb complex to the nascent viral transcript, where it coalesces with other factors to form a superelongation complex (SEC) (Fig. 1*A*) (10, 15, 16, 17, 18, 19). CDK9 within the SEC phosphorylates host RNA polymerase II, releasing it from a paused state to transcribe full-length viral transcripts (6, 7). This pathway highlights distinct RNA-protein molecular recognition events that serve as potential targets to upend viral transcription.

**Figure 1:**
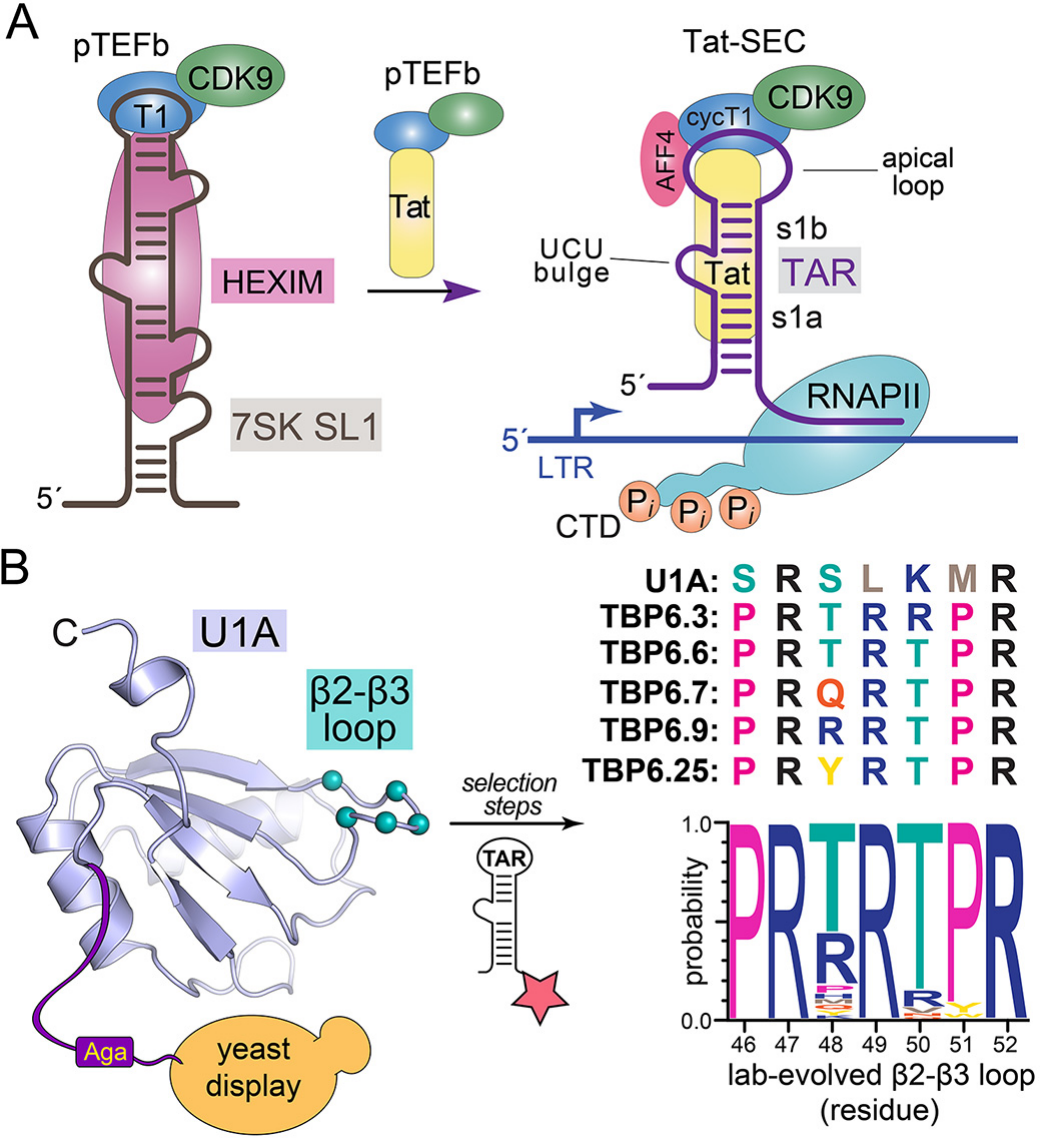
**Schematic diagram of Tat interactions with human 7SK and HIV TAR leading to the SEC, and overview of the selection process that produced lab-evolved TBPs.***A*, *left*, *cartoon* depicting the inactive pTEFb complex sequestered by the host 7SK small-nuclear ribonucleoprotein (*7SK RNP*) complex that includes protein HEXIM. The ARM of HIV-1 Tat protein mimics that of HEXIM, displacing the pTEFb complex, which contains CycT1 and CDK9. *Right arrow*, Tat transfers the pTEFb complex by direct interaction with HIV-1 TAR at the 5′-LTR. This complex subsequently forms the SEC. Transcription elongation of viral mRNA is then activated by CDK9-mediated phosphorylation of the RNA polymerase II C-terminal domain (6, 7). *B*, *left*, crystal structure of U1A from the protein-RNA complex (PDB entry 1URN) showing amino acids that were diversified in the β2-β3 loop based on their contact with hpII RNA (8). Selection was performed by yeast display and cell sorting based on binding to fluorescently labeled TAR (*star*). The U1A fold exhibits a βαββαβα topology typical of the single-stranded RRM (9). *Right*, sequences of the β2-β3 loop from U1A and lab-evolved TBPs of this investigation. A Weblogo analysis highlights the consensus of the lab-evolved β2-β3 loop. Adapted from Ref. 8.

The precise role of arginine in Tat-mediated RNA recognition came into focus only recently based on the high-resolution NMR analysis of an HIV Tat-ARM in complex with human 7SK RNA and HIV TAR RNA (19). When bound to host 7SK, Tat adopts a bent conformation wherein a cluster of three arginines specifically recognizes the Hoogsteen edges of guanine nucleobases. Conversely, the Tat-ARM adopts a more linear conformation that uses two arginines to recognize the Hoogsteen edges of TAR nucleobases. Remarkably, these different modes of base recognition use distinctive arginines from the same Tat peptide. Additional affinity arises from hydrogen bonding, cation-π stacking, and electrostatic interactions that give rise to nanomolar affinity of the Tat ARM for each of these distinct human and viral RNAs (19).

The essentiality of the Tat-TAR complex has prompted researchers to target TAR with inhibitors that block Tat binding (11, 12). Such molecules have been imbued with ARM-like properties, including arginine, guanidinium, or amiloride groups (13, 14, 20, 21). Proof-of-concept for this molecular-mimicry approach was exemplified by a family of cyclic peptides that emulate the BIV Tat-ARM, which folds as a β-hairpin (22, 23, 24, 25). These inhibitors recognize HIV TAR with *K_D_* values in the nanomolar to picomolar range, while yielding *K_i_* values of ∼40 μm in antiviral assays (25). Importantly, NMR analysis of an ultrahigh-affinity inhibitor from this family revealed TAR recognition at two guanine nucleobases by specific arginines (25), demonstrating parallels to the natural modes of TAR and 7SK recognition by the Tat ARM (12). These observations pose the question of whether peptide inhibitors could be developed that use three or more arginines to target the Hoogsteen edges of conserved guanines in HIV TAR, thereby imparting even greater specificity.

Recently a new class of arginine-dependent, protein-based TAR inhibitors was introduced that used a lab-evolution approach (Fig. 1*B*). Starting from the high-affinity interaction between the U1A RNA recognition motif (RRM) and U1 small nuclear RNA (26), specific RNA-interacting amino acids were subjected to saturation mutagenesis (8, 27). Using yeast display and multiple rounds of selection, a new class of TAR-binding proteins (TBPs) was identified that bind TAR with nanomolar affinity (Fig. 1*B*) (8, 27). Our laboratory determined the co-crystal structure of the TAR-TBP variant 6.7 complex (*i.e.* TBP6.7), which revealed how three arginines within the lab-evolved β2-β3 loop recognize TAR at three conserved guanine nucleobases (28). This structure along with supporting biochemical data further suggested that the β2-β3 loop was necessary and sufficient for TAR binding. Accordingly, a 20-amino acid stapled peptide was synthesized that bound to TAR with a *K_D_* of 1.8 ± 0.5 μm (28). In contrast to previous work, this result demonstrated that three arginines were operative in TAR recognition. Intriguingly, multiple other TBP variants were selected with three or four arginines in the β2-β3 loop (29) (Fig. 1*B*). Variations in the number and spacing of these arginines suggested the possibility that some variants use all four arginines to recognize TAR, that new modes of recognition—distinct from TBP6.7—were possible, and that some sequences and arginine spacings would be superior TAR binders compared with others.

To address these questions and provide greater insight into TAR molecular recognition, we used isothermal titration calorimetry (ITC) to quantify how specific arginine compositions and spacings in the β2-β3 loop affect TAR binding. We evaluated TBP variants 6.3, 6.9, 6.6, 6.25 (Fig. 1*B*) and a double mutant of TBP6.7 (Q48R/T50R) that contains five loop arginines. We then determined the corresponding co-crystal structures of these variants in complex with TAR. The results reveal how sequence differences in the β2-β3 loop cause structural changes that alter TAR recognition and stabilization of the β2-β3 loop. Based on these observations, we designed short macrocyclic peptides that mimic the β2-β3 loop and contain three or four arginines within a small (11-mer) cyclic peptide scaffold. Importantly, these compounds were determined to interact with TAR and to block Tat ARM binding to TAR, enabling the discovery of a new inhibitor of the TAR-Tat interaction. Our findings are discussed in the context of how diverse ARM interactions recognize RNAs and the principles of molecular recognition used by other macrocyclic peptides known to target the TAR-Tat interface.

## Results

### Structure determination of TAR-TBP co-crystal structures and quality control

To gain insight into TAR recognition by a family of lab-evolved proteins, we determined the co-crystal structures of TBP variants 6.3, 6.6, and 6.9 and a double mutant of TBP6.7, Q48R/T50R. We chose these proteins because they differ in their RNA-binding sequences within the lab-evolved β2-β3 loop, which was shown previously to be essential for TAR recognition (Fig. 1*B*) (28). The diffraction of samples varied between 1.71 and 3.10 Å resolution (Table 1), and each structure is of acceptable quality based upon *R*_work_ values ranging from 19.1 to 22.8% and *R*_free_ values no more than 6% above *R*_work_, indicating that the models are not overfit (33). No breaks were observed in electron density maps for TBP main chains, and the complete nucleotide sequence was modeled for each TAR 27-mer. Each structure also exhibited acceptable geometry (root mean square deviation (r.m.s.d.) bonds <0.01 Å and angles <1.3°) with clash scores <1 and MolProbity scores between 0.58 and 0.90 (32). The quality of these structures and our ability to discern new chemical interactions in electron density maps (Fig. S1) prompted us to proceed with structure and affinity analyses.

**Table 1:** X-ray data collection and refinement statistics

Sample	TAR-TBP6.9	TAR-TBP6.7 Q48R/T50R	TAR-TBP6.6	TAR-TBP6.3
**Data collection**^a^				
PDB code	6XH0	6XH1	6XH2	6XH3
Wavelength (Å)	0.98	0.98	0.98	0.98
Resolution range (Å)	38.97–3.10 (3.31–3.10)	39.85–2.60 (2.72–2.60)	39.91–1.71 (1.74–1.71)	39.12–2.35 (2.43–2.35)
Space group	*P*4_3_2_1_2	*P*4_3_2_1_2	*P*4_3_2_1_2	*P*4_3_2_1_2
*a = b, c* (Å)	40.5, 286.4	40.2, 293.9	40.3, 288.7	40.6, 292.0
α = β = γ (°)	90	90	90	90
Total observations	57,567	61,674	222,548	78,357
Multiplicity	11.5 (12.1)	7.9 (7.9)	8.2 (7.5)	7.1 (5.6)
Completeness (%)	99.7 (100)	96.5 (97.7)	99.2 (88.4)	98.9 (91.5)
Mean *I*/σ(*I*)	8.2 (1.6)	7.7 (2.4)	10.7 (1.5)	7.6 (1.6)
*R*_p.i.m._ (%)^b^	7.1 (44.7)	6.5 (55.0)	3.3 (38.5)	6.2 (43.9)
CC_½_^c^	0.998 (0.743)	0.986 (0.403)	0.994 (0.635)	0.975 (0.595)
**Refinement**				
No. of reflections (work/test)	4,920/493	7,660/561	26,782/2,000	11,000/1,433
*R*_work_/*R*_free_ (%)^d^	22.8/28.8	21.6/25.5	19.1/20.9	21.3/25.3
No. of atoms				
Protein	709	707	770	722
RNA	572	572	572	572
Solvent	3	43	98	65
r.m.s.d. (bonds)	0.002	0.002	0.010	0.001
r.m.s.d. (angles)	0.39	0.41	1.25	0.42
Ramachandran favored (%)	97.6	98.8	100	98.8
Ramachandran allowed (%)	2.4	1.2	0.0	1.2
Ramachandran outliers (%)	0.0	0.0	0.0	0.0
Coord. error^e^	0.51	0.34	0.16	0.33
Clashscore^e^	0.0	0.43	0.82	0.43
Molprobity score	0.58	0.65	0.90	0.73
Average *B*-factor				
Protein (Å^2^)	73	42	39	42
RNA (Å^2^)	83	49	45	50
Waters (Å^2^)	65	38	43	43


a Statistics for the highest-resolution shell are shown in parentheses.

b precision-indicating merging R-value = $\frac{\sum_{hkl}\sqrt{\frac{1}{N-1}}\sum_{i=1}^{N}|I\left(hkl\right)-〈I(hkl)〉|}{\sum_{hkl}^{}\sum_{i=1}^{N}I(hkl)}$, where *N* is the redundancy of the data and $〈I(hkl)〉$ is the average intensity (30). This metric corrects for data redundancy and replaces the outdated *R*_merge_ statistic that fails to correct for redundancy, thereby incorrectly making redundant data appear poorer in quality.

c CC_½_, Pearson correlation coefficient between intensities of random half-data set (31).

d *R*_work_ = Σ*_hkl_*‖*F*_obs_(*hkl*)| − |*F*_calc_(*hkl*)‖)/Σ*_hkl_*|*F*_obs_(*hkl*)| for the working set of reflections, and *R*_free_ is defined as *R*_work_ for ∼10% of the reflections excluded from the refinement. All data from the available resolution ranges were used in the refinement.

e Calculated using the program Molprobity (32).

### TAR-TBP co-crystal structures adopt similar folds and conformations

**Figure 2:**
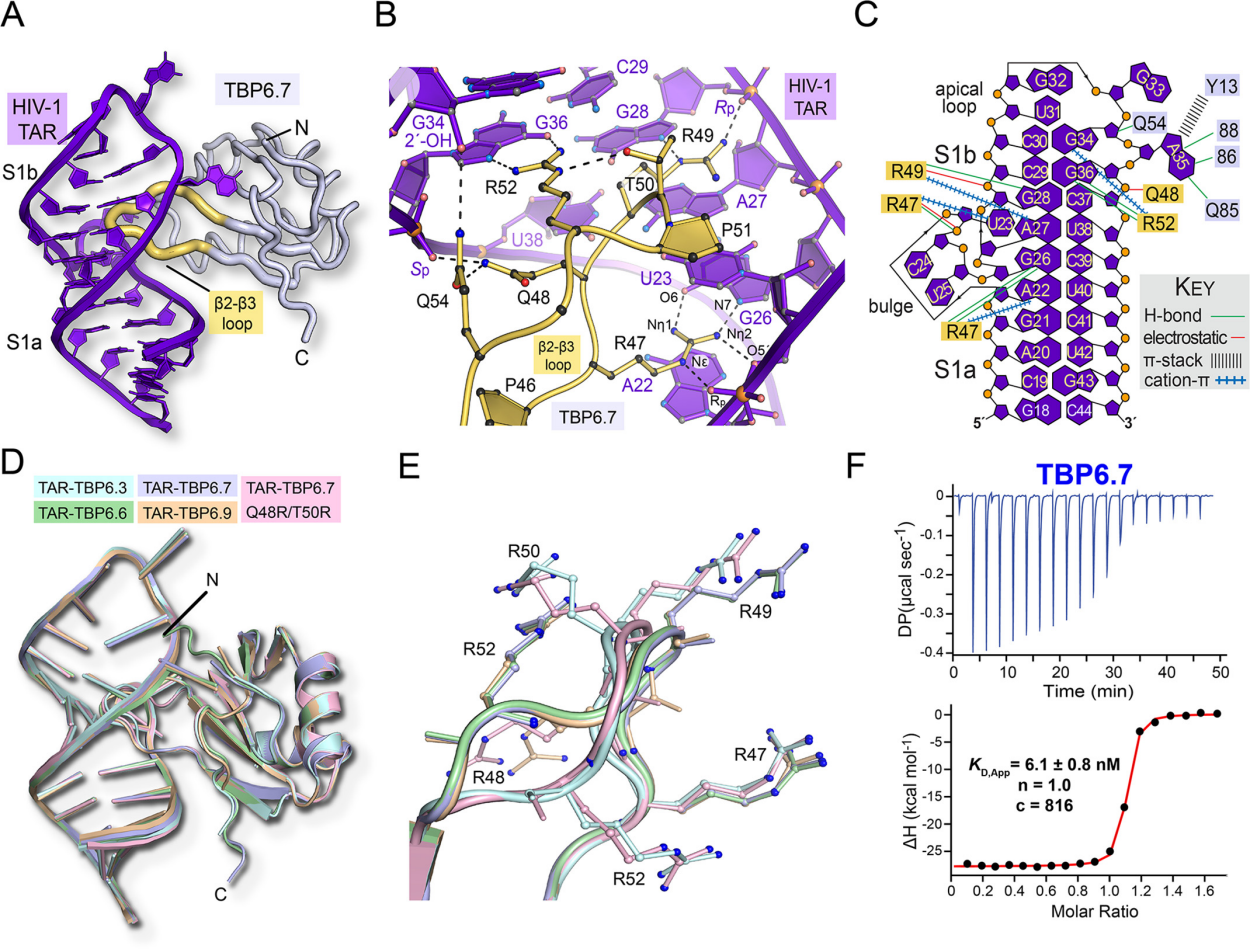
**Structural overview of lab-evolved protein TBP6.7, superpositions of HIV TAR-TBP co-crystal structures of this investigation, and TAR binding to TBP6.7.***A*, *ribbon diagram* depicting the overall fold of the TAR-TBP6.7 complex reveals entry of the β2-β3 loop into the major groove (PDB entry 6CMN) (28). *B*, *close-up view* of the lab-evolved β2-β3 loop of TBP6.7 showing TAR readout that includes Arg-47, which reads the Hoogsteen edge of Gua26 and the Uri23 backbone; Arg-49, which recognizes N7 of Gua28 and the phosphate backbone; and Arg-52, which reads the Hoogsteen edge of Gua36. Here and elsewhere, putative interactions are shown by *dashed lines*. *C*, overview of chemical interactions between the TAR-TBP6.7 complex that are representative of the various RNA-protein interactions of other TBPs in the current investigation. *D*, pairwise all-atom superposition of the co-crystal structures of this investigation upon the TAR-TBP6.7 complex. The average r.m.s.d. was 0.43 Å. The overall three-dimensional fold of each TBP is similar to TBP6.7. TAR RNA also reveals structurally similar details (Fig. S2). *E*, *close-up view* of the superimposed β2-β3 loops from *D*. Arginine placement affects the loop conformation and the mode of TAR recognition. The average loop r.m.s.d. was 0.78 Å. *F*, representative ITC thermogram of TBP6.7 titrated into TAR. The apparent equilibrium dissociation constant (*K_D_*) is shown, along with the stoichiometry (*n*), and the *c* value to indicate the quality of the binding model fit (34). Here and elsewhere, the representative single-run ITC parameters shown on thermograms differ from Table 2, which reports average values from duplicate titrations.

The co-crystal structure of HIV-1 TAR in complex with TBP6.7 was determined previously in our laboratory to 1.80 Å resolution (28). This work revealed a novel mode of dsRNA recognition in which the lab-evolved β2-β3 loop penetrates deeply into the TAR major groove at a central UCU bulge (Fig. 2*A*). This structure provides a benchmark for TAR-TBP–mediated interactions, which cluster in the TAR major groove and acquire specificity through Arg-47–, Arg-49–, and Arg-52–mediated readout of Gua26, Gua28, and Gua36 (Fig. 2*B*). Additional amino acids of the β2-β3 loop, such as Gln-48, recognize the phosphate backbone or form intrapeptide hydrogen bonds, such as Thr-50 and Gln-54, supporting a stable loop conformation (Figs. 2, *B* and *C*).

In the context of each new complex, the overall conformation of each TBP appears almost unchanged compared with TBP6.7 (Fig. 2*D*). Conformational similarity was confirmed by the average r.m.s.d. of 0.43 ± 0.10 Å obtained from a pairwise superposition of all nonhydrogen atoms from each TBP upon TBP6.7. Like TBP6.7, the β2-β3 loop of each variant reaches far into the major groove (Fig. 2*D*). Despite changes in sequence, each loop exhibited a similar overall conformation as indicated by an average pairwise r.m.s.d. of 0.78 ± 0.36 Å (Fig. 2*E*), where differences are most notable at positions 49, 50, and 52. The latter r.m.s.d. is larger than the average coordinate error of 0.34 ± 0.07 Å measured from all structures (Table 1). In contrast, a similar superposition with the parental U1A β2-β3 loop showed an average pairwise r.m.s.d. of 2.60 Å. Hence, the β2-β3 loop undergoes a significant conformational change as a result of laboratory evolution and TAR binding, but the conformations adopted by individual TBPs are relatively unaltered when bound to TAR. Notably, none of the lab-evolved C-terminal residues were observed to participate in TAR binding, in agreement with our previous findings for TBP6.7 (28).

### TAR RNA in complex with TBPs retains hallmark features of the ligand-bound state

HIV TAR exhibits significant conformational dynamics and flexibility in solution (12). For these reasons, it is informative to consider how TAR changed its conformation in response to binding by various TBPs. The average pairwise r.m.s.d. of all TAR molecules of this investigation superimposed onto the TAR-TBP6.7 complex was 0.53 ± 0.13 Å. Accordingly, all RNA molecules reveal common structural traits, including A-form helical stems s1a and s1b separated by the Uri23·Ade27-Uri38 major-groove base triple (Fig. 2 (*A* and *C*) and Fig. S2A). The latter long-range interaction is a hallmark of the ligand-bound conformation in solution (24, 35). A nearby central bulge flanks the base triple and is observed in all TAR-TBP complexes. Each structure reveals Cyt24 and Uri25 protruding away from the helical core (Fig. S2B). The pinnacle of each TAR hairpin features an apical hexaloop wherein Uri31 and Gua32 stack atop Cyt30 (Fig. S2C). The latter base forms a canonical pair with Gua34, whereas Gua33 and Ade35 bulge away from the hexaloop. This apical loop conformation closely resembles the major conformation in solution, which is in exchange with a minor excited state (36). These features agree well with those observed in the previous TAR-TBP6.7 complex (28), despite the notorious flexibility of HIV-1 TAR RNA in the ligand-bound state (19, 29, 35, 37).

### TBPs with disparate arginine content and spacing exhibit unique binding signatures

Generation of the TBP family entailed a lab-evolution process that yielded multiple proteins that bind TAR and show three or four arginines at distinct locations within the β2-β3 loop (Fig. 1*B*) (8). These results raised the question of whether variations in arginine composition and placement in the β2-β3 loop could substantially alter affinity for TAR. Although previous work verified binding interactions between specific TBPs and TAR, the approach did not measure equilibrium binding constants for each TBP variant (8).

To quantify the equilibrium binding constants of specific TAR-TBP complexes, we expressed and purified TBP variants 6.3, 6.6, 6.9, and 6.25 for ITC analysis. As a basis for comparisons (*i.e. K*_rel_ in Table 2) we used the previously characterized TAR-TBP6.7 interaction, due to its known structure and because it blocked Tat-dependent transcription in HeLa nuclear lysate (8, 28). We first measured TBP6.7 binding to TAR and observed a *K_D_* of 5.3 ± 0.9 nm, an unfavorable entropic contribution (−*T*Δ*S* of 13.9 ± 2.9 kcal mol^−1^) and a favorable enthalpic contribution that drives binding (Δ*H* of −25.0 ± 2.9 kcal mol^−1^ (Fig. 2*F* and Table 2). These measurements are consistent and comparable with our prior ITC analysis (28).

**Table 2:** Average thermodynamic parameters for TAR binding by TBPs and peptides at 20 °C

Sample	*K_D_*	Sites (*n*)	Δ*Η*^°^	−*T*Δ*S*^°^	Δ*G*^°^	ΔΔ*G*^a^	*K*_rel_^b^	β2-β3 loop (residues 47–52)
	*nm*		*kca/mol*	*kcal/mol*	*kcal/mol*	*kcal/mol*		
TBP6.3	45.2 ± 3.4	1.0	−18.4 ± 1.7	8.5 ± 1.8	−9.9 ± 0.1	1.2	8.5	RTRRPR
TBP6.6	4.2 ± 0.3	1.0	−24.8 ± 0.5	13.6 ± 0.6	−11.3 ± 0.1	−0.2	0.8	RTRTPR
TBP6.7	5.3 ± 0.9	1.0	−25.0 ± 2.9	13.9 ± 2.9	−11.1 ± 0.1	0.0	1.0	RQRTPR
TBP6.9	3.0 ± 0.3	1.0	−22.3 ± 1.6	11.6 ± 1.4	−11.5 ± 0.1	−0.4	0.6	RRRTPR
TBP6.25	71.1 ± 2.0	1.0	−15.3 ± 2.0	5.7 ± 2.0	−9.6 ± 0.1	1.5	13.4	RYRTPR
Q48R/T50R^c^	27.8 ± 6.5	1.0	−16.6 ± 2.8	6.4 ± 2.6	−10.2 ± 0.2	0.9	5.2	RRRRPR
Tat ARM	135 ± 31	2.0	−4.5 ± 0.6	−4.8 ± 0.7	−9.2 ± 0.1	NA^d^	NA	—e
TB-CP-6.9a	5.3 ± 0.2	1.0	−7.2 ± 0.2	0.1 ± 0.2	−7.1 ± 0.02	NA	NA	RRRTPR


a The difference of (Δ*G*°_mutant_ or Δ*G*°_variant_) − Δ*G*°_TBP6.7_.

b Defined as the ratio of [mutant *K_D_*]/[WT *K_D_*] TBP6.7.

c A β2-β3 loop variant generated for this study based on the TBP6.7 protein.

d NA, not applicable.

e The Tat ARM sequence is GISYGRKKRRQRRRAHQ.

**Figure 3:**
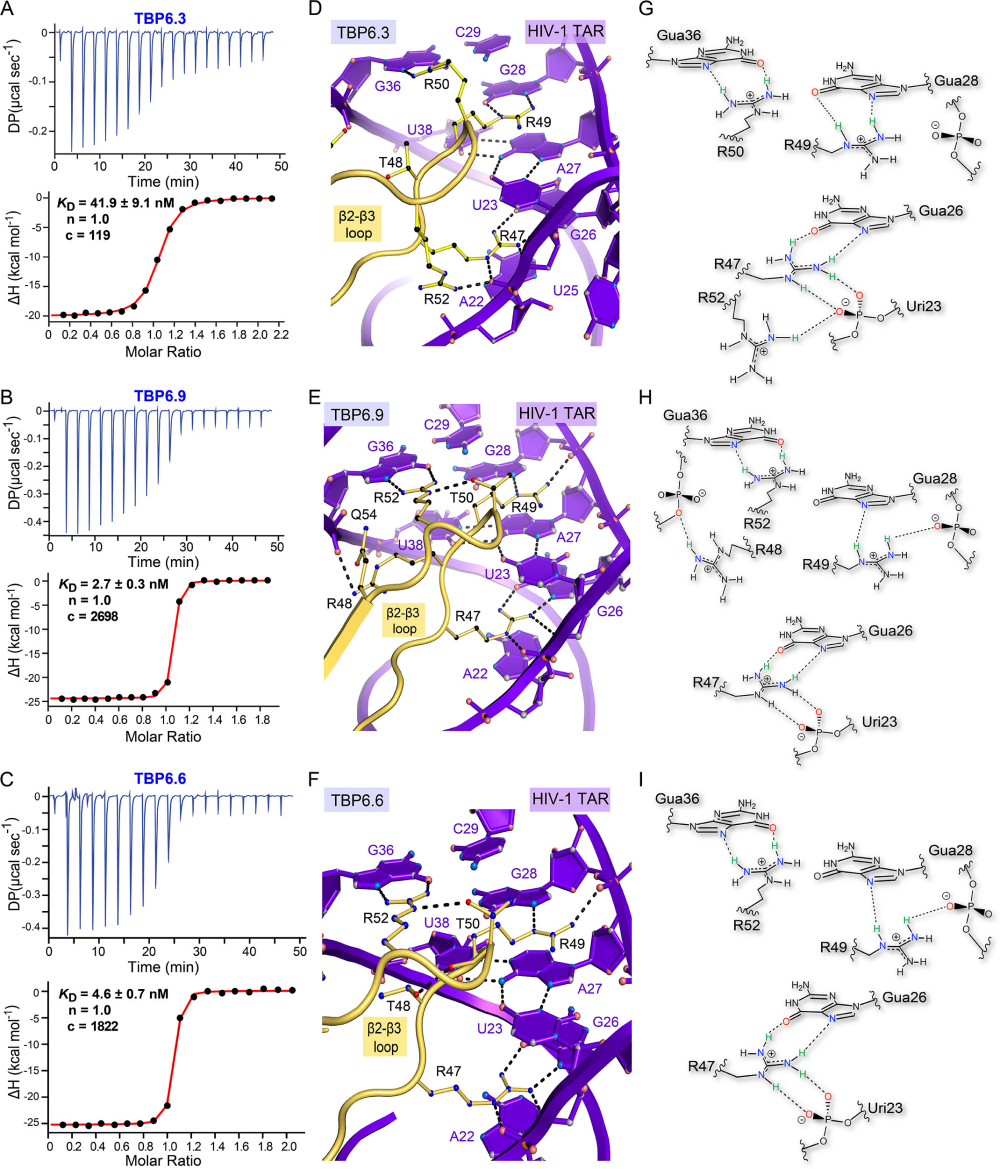
**Affinity analysis of TBP variants for HIV-1 TAR and *close-up views* of TAR-TBP co-crystal structures revealing arginine-mediated RNA recognition.***A*, representative ITC thermogram of TBP6.3 titrated into TAR. *B*, representative ITC thermogram of TBP6.9 titrated into TAR. *C*, representative ITC thermogram of TBP6.6 titrated into TAR. *D*, *close-up view* of the lab-evolved β2-β3 loop of TBP6.3 showing TAR readout by four arginines. Arg-47 and Arg-49 retain interactions similar to TBP6.7 (Fig. 2, *B* and *C*), but Arg-52 is displaced by Arg-50 to recognize Gua36. As a result, Arg-52 now binds the backbone pro-*R*_p_ oxygen of Uri23. *E*, *close-up view* of the lab-evolved β2-β3 loop of TBP6.9 showing TAR readout by four arginines, including three consecutive arginines. Arg-48 interacts with the backbone at the pro-*S*_p_ oxygen of Gua36, similar to Gln-48 of TBP6.7, whereas Arg-47, Arg-49, and Arg-52 retain Hoogsteen-edge readout similar to TBP6.7 (Fig. 2, *B* and *C*). *F*, *close-up view* of the lab-evolved β2-β3 loop of TBP6.6 showing TAR readout by three arginines. The mode of interaction is comparable with TBP6.7 (Fig. 2*B*), except that Thr-48 and Thr-50 engage in stabilizing side chain-to-backbone interactions. *G*, schematic diagram depicting arginine interactions between TBP6.3 and TAR in *D*. *H*, schematic diagram depicting arginine interactions between TBP6.9 and TAR in *E*. *I*, schematic diagram depicting arginine interactions between TBP6.6 and TAR in *F*.

**Figure 4:**
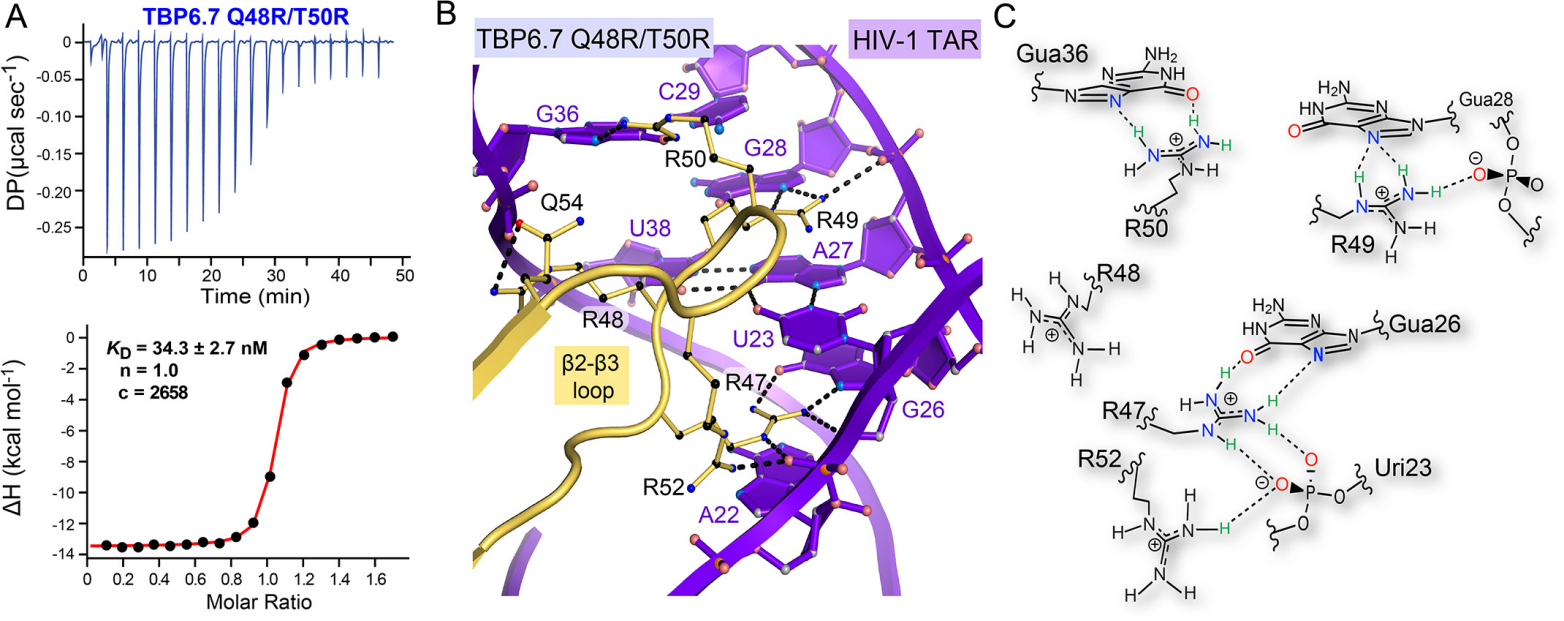
**Affinity analysis of five-arginine mutant TBP6.7 Q48R/T50R for TAR RNA and *close-up view* of the corresponding co-crystal structure.***A*, representative thermogram of TBP6.7 Q48R/T50R titrated into TAR. *B*, *close-up view* of variant TBP6.7 Q48R/T50R showing readout of TAR using four arginines. Arg-47 retains interactions similar to TBP6.7 (Fig. 2, *B* and *C*). Arg-48 interacts with the pro-*S*_p_ nonbridging oxygen of Gua36, as seen for TBP6.9 (Fig. 3, *E* and *H*). Nε and of Nη2 of Arg-49 contact N7 and the pro-*R*_p_ oxygen of Gua28. Arg-50 recognizes the Hoogsteen edge of Gua36, displacing Arg-52, which salt-bridges to the pro*-R*_p_ oxygen of Uri23. The former and latter interactions parallel Arg-50 of TBP6.3 and Arg-52 of TBP6.9 (Fig. 3 (*panels d* and *g* and *panels e* and *h*)). *C*, schematic diagram depicting arginine interactions from *B* and their interactions in the co-crystal structure. Like all other TBPs, a trio of arginines recognizes the Hoogsteen edges of conserved guanines in the major groove. There are no apparent intrapeptide stabilization interactions.

Despite sequence differences relative to TBP6.7, each new TBP was observed to bind similarly to TAR with 1:1 binding stoichiometry, *K_D_* values ranging from 3.0 ± 0.3 to 71.1 ± 2.0 nm, an unfavorable entropy (−*T*Δ*S* ranging from 5.7 ± 2.0 to 13.6 ± 0.6 kcal mol^−1^), and a binding reaction driven by enthalpy (ranging from −24.8 ± 0.5 to −15.3 ± 2.0 kcal mol^−1^) (Figs. 3 (*A–C*) and 4*A* and Table 2). Tangible differences in *K*_rel_ by >13-fold (Table 2) and changes in other thermodynamic signatures prompted us to systematically consider how arginine content and placement in various β2-β3 loops affected TAR binding in terms of molecular recognition.

### A fourth arginine at position 48 favors binding, but placement at position 50 is deleterious

To examine the role of arginine placement in TAR recognition, we first considered TBP6.3, which differs from TBP6.7 by the change of Q48T and T50R (Fig. 1*B*). ITC analysis showed a *K_D_* of 45.2 ± 3.4 nm for TBP6.3, representing a ΔΔ*G*° of +1.2 kcal mol^−1^ compared with TBP6.7—signifying a less favorable binding equivalent to ∼2 hydrogen bonds (*K*_rel_ of 8-fold) (Fig. 3*A* and Table 2). A Weblogo analysis of >70 TBPs (8) revealed that Thr-48 and Arg-50 correspond to the major and minor amino acids found in the β2-β3 loop consensus sequence (Fig. 1*B*). The co-crystal structure of the TAR-TBP6.7 complex revealed that Gln-48 interacts with the TAR backbone, whereas Thr-50 stabilizes the β2-β3 loop by hydrogen bonding to Arg-52 (28) (Fig. 2*B*). However, we observed that Arg-50 of TBP6.3 displaced Arg-52, usurping its role in Gua36 recognition. Arg-52 adopted a new conformation that protrudes into the TAR bulge to interact with the backbone at the pro-*R*_p_ oxygen of Uri23 (Fig. 3, *D* and *G*). Thr-48 had no deleterious effects except that its side chain is too short to hydrogen-bond with the phosphate backbone as observed for Gln-48 in TBP6.7 (Figs. 2*B* and 3*D*).

Although TBP6.3 contains four arginines, this variant exhibited 8.5-fold weaker binding than TBP6.7 (Table 2), which has three β2-β3 loop arginines (Fig. 1*B*). This observation prompted us to investigate TBP6.9 because it is identical to TBP6.7, except that position 48 is replaced by arginine. Unexpectedly, ITC revealed that TBP6.9 bound to TAR with a *K_D_* of 3.0 ± 0.3 nm and ΔΔ*G*° of −0.4 kcal mol^−1^, indicating a nearly equivalent interaction energy compared with TBP6.7 and a slightly more favorable *K*_rel_ of 0.6 (Table 2). As both TBP6.3 and TBP6.9 evolved with four arginines in their β2-β3 loops, we expected to see novel arginine interactions relative to TBP6.3 or TBP6.7. However, the TAR-TBP6.9 co-crystal instead revealed only a subtle variation of the fundamental set of interactions first observed for three-arginine recognition of TAR by TBP6.7. Specifically, the Arg-48 guanidinium group of TBP6.9 interacts with the backbone at the pro-*S*_p_ oxygen of Gua36 in a manner analogous to Gln-48 of TBP6.7; simultaneously, Arg-47, Arg-49, and Arg-52 retained interactions with conserved major-groove guanine bases (Figs. 2*B* and 3 (*E* and *H*)).

Although TBP6.3 contains four arginines like TBP6.9, the former exhibits two suboptimal interactions. Namely, the Arg-50 guanidinium is not co-planar with the Gua36 base, yielding a weak inclined hydrogen bond interaction to the base Hoogsteen edge. Similarly, accommodation of Arg-50 in place of Arg-52 pulls nearby Arg-49 away from the phosphate backbone such that the Arg-49 guanidium group hydrogen-bonds to O6 and N7 of Gua28 (Fig. 3, *D* and *G*) rather than N7 and the Gua28 phosphate—as observed in TBP6.9 (Fig. 3, *E* and *H*). Differences between TBP6.3 and TBP6.9 underscore two important observations: (i) optimal arginine composition and placement supports TAR recognition through distinct sets of interactions with the Hoogsteen edges of guanine bases and the phosphate backbone, and (ii) intramolecular hydrogen bond interactions within the β2-β3 loop contribute peptide stability for the TAR major-groove region.

To further test the latter observations, we analyzed variant TBP6.6, which possesses threonine at positions 48 and 50 (Fig. 1*B*). ITC revealed high-affinity TAR binding based on a *K_D_* of 4.2 ± 0.3 nm (Fig. 3*C* and Table 2). Despite only three arginines, we hypothesized that this variant would possess strong affinity comparable with TBP6.7 or TBP6.9 because each threonine has the ability to form hydrogen bonds that stabilize the β2-β3 loop backbone. As expected, the co-crystal structure revealed an interaction between Oγ1 of Thr-48 and the carbonyl oxygen of Thr-50 (Fig. 3 (*F* and *I*) and Fig. S3A), as observed for TBP6.7 and TBP6.9. A second interaction is also shared among TBP6.7, TBP6.9, and TBP6.6. Each protein makes a hydrogen bond between Oγ1 of Thr-50 and Nε of Arg-52, stabilizing a favorable arginine rotamer for guanine recognition (Figs. 2*B* and 3 (*E* and *F*) and Fig. S3B). In contrast, TBP6.3 cannot form this interaction because Thr-50 was replaced by Arg-50 (Figs. 1*B* and 3*D*). As such, backbone stabilization interactions are suboptimal for this variant.

A main take-home message for TBP6.6 is that stabilization of the β2-β3 loop by threonine-mediated intramolecular hydrogen bonding is favorable. TBP6.6's ΔΔ*G*° of −0.2 kcal mol^−1^ is slightly more favorable compared with TBP6.7, and its Δ*G*° is equivalent to TBP6.9 (Table 2). These observations provide a rationale for the preference of threonine at positions 48 and 50 and how glutamine and arginine are tolerated at position 48, whereas arginine is not well-tolerated at position 50 (Fig. 1*B*).

### An aromatic group between arginines is detrimental for β2-β3 loop binding to TAR

RRMs such as U1A, the TBP progenitor (Fig. 1*B*), use aromatic residues to recognize their single-stranded RNA targets (28, 38). This observation led us to explore TBP6.25, which has a tyrosine at position 48 — an amino acid represented only sparsely in the β2-β3 loop consensus. Otherwise, the TBP6.25 sequence is identical to TBP6.6 and TBP6.7. ITC of the 6.25 variant revealed a *K_D_* of 71.1 ± 2.0 nm and a ΔΔ*G*° of +1.5 kcal mol^−1^ (Fig. S3C and Table 2). This free energy change corresponds to 13-fold weaker affinity for TAR relative to TBP6.7 and ∼2-fold weaker binding than TBP6.3. This reduced affinity is notable because the latter variant exhibits two amino acid differences in the β2-β3 loop, whereas TBP6.25 shows only the Tyr-48 change. Based on the observed roles of position 48 in phosphate or backbone hydrogen bonding (*i.e.* TBP6.6, TBP6.7, and TBP6.9), it seems plausible that Tyr-48 cannot engage in either interaction due to the bulk of the phenolic side chain. Unfortunately, our efforts to produce X-ray diffraction quality crystals of the TAR-TBP6.25 complex were unsuccessful. As such, we turned to our previous analysis in which we explored the role of position 48. We showed that the Q48A mutation in the context of TBP6.7 produced a ΔΔ*G*° of +0.5 kcal mol^−1^ corresponding to a *K*_rel_ of 2.2 (28). In contrast, the Q48T mutant produced a *K*_rel_ of 0.8 and a ΔΔ*G*° = −0.2 kcal mol^−1^, as inferred from TBP6.6 (Table 2). This analysis suggests that TBP6.25 is significantly more disruptive than simply breaking a hydrogen bond with the phosphate backbone, such as Q48A, or disrupting an intramolecular hydrogen bond, such as Q48T. Structures of TBP6.6 and TBP6.9 (Fig. S3, A and B) suggest that Tyr-48 would interfere with Arg-52–mediated recognition of the Gua36 Hoogsteen edge. For comparison, complete disruption of the latter interaction yielded a *K*_rel_ of 116 and a ΔΔ*G*° = 2.8 kcal mol^−1^, which was measured for the R52A mutation to TBP6.7 (28). Accordingly, TBP6.25 likely ablates stabilizing interactions with the phosphate and peptide backbones while partially compromising nearby nucleobase readout.

Collectively, the ITC results reveal that placement of arginine at position 48 can be favorable in the context of existing arginines at positions 47, 49, and 52—as seen for TBP6.9. However, placement of a bulky aromatic side chain at position 48 was unfavorable for TBP6.25. In contrast, arginine at position 50—as seen for TBP6.3—was somewhat unfavorable by comparison. These observations highlight how arginine placement in the β2-β3 loop can fine-tune affinity for TAR binding.

### Five arginines in the β2-β3 loop do not promote readout of more nucleobases

We noted that a penta-arginine sequence was not among those selected during lab-evolution of the TBP β2-β3 loop (Fig. 1*B*). To probe this outcome, we integrated five arginines into the β2-β3 loop of TBP6.7 as the Q48R/T50R mutant. We rationalized that a β2-β3 loop with five arginines might rearrange its side chains in a manner similar to TBP6.3 to accommodate additional guanidinium groups in the major groove. ITC analysis revealed that the new variant recognizes TAR with *K_D_* of 27.8 ± 6.5 nm and a ΔΔ*G*° of +0.9 kcal mol^−1^ (Fig. 4*A* and Table 2), corresponding to a *K*_rel_ ∼5-fold weaker in binding than WT TBP6.7.

To understand the molecular basis of these thermodynamic properties, we next determined the co-crystal structure of the TAR-TBP6.7 Q48R/T50R complex. Again, the structure indicated that only three arginines could bind simultaneously to the Hoogsteen edges of guanine bases in the context of the β2-β3 loop (Fig. 4*B*). This variant makes contacts to TAR in a manner analogous to both TBP6.3 and TBP6.9. Specifically, Arg-52 interacts with the pro-*R*_p_ nonbridging phosphate oxygen of Uri23 when displaced by Arg-50—as in TBP6.3. Arg-48 interacts with the pro-*S*_p_ nonbridging phosphate oxygen of Gua36 as in TBP6.9. Arg-47, Arg-49, and Arg-50 retain their interactions with major-groove guanines as seen in TBP6.3 (Fig. 4*C*). No apparent backbone stabilization interactions form between β2-β3 loop residues.

Analysis of the TBP6.7 Q48R/T50R double mutant highlights important aspects of TAR recognition. First, the addition of extra arginines in the β2-β3 loop—beyond the initial three—did not promote additional interactions at the Hoogsteen edges of guanine bases. Second, the need to maintain charge separation appears to have influenced arginine locations and rotamer conformations in the β2-β3 loop. Third, residues that fill the gaps between base-recognition arginines were of benefit when they stabilized the polypeptide via intramolecular hydrogen bonds.

### Cyclic peptides derived from TBP6.7 and TBP6.9 show different levels of TAR binding

So far, our structural and binding analyses defined the β2-β3 loop as a critical motif for recognition of TAR, suggesting the possibility to mimic this interaction by means of a small cyclic peptide comprising primarily loop residues. Conformational restriction of protein recognition motifs has proved to be an attractive strategy to develop inhibitors of protein-protein interactions (28, 39, 40). Using the results from our TBP analysis herein, we designed a small cyclic peptide, called TB-CP-6.7a (*i.e.* TAR-binding cyclic peptide from TBP6.7a in which appended letter “a” represents a methylene linker). The peptide encompasses the ^46^PRQRTPRGQ^54^ sequence corresponding to the β2-β3 loop of TBP6.7 (Fig. S4A). This peptide sequence was cyclized by means of two terminal cysteines cross-linked via a methylene bridge to produce a stable linkage (41, 42) between the ends of strands β2 and β3. For comparison, a linear peptide (*i.e.* TB-LP-6.7) was also prepared in which the cysteines were alkylated with iodoacetamide (Fig. S4B). Both peptides were prepared via solid-phase peptide synthesis and characterized by MS and analytical HPLC (Fig. S4, A and B).

**Figure 5:**
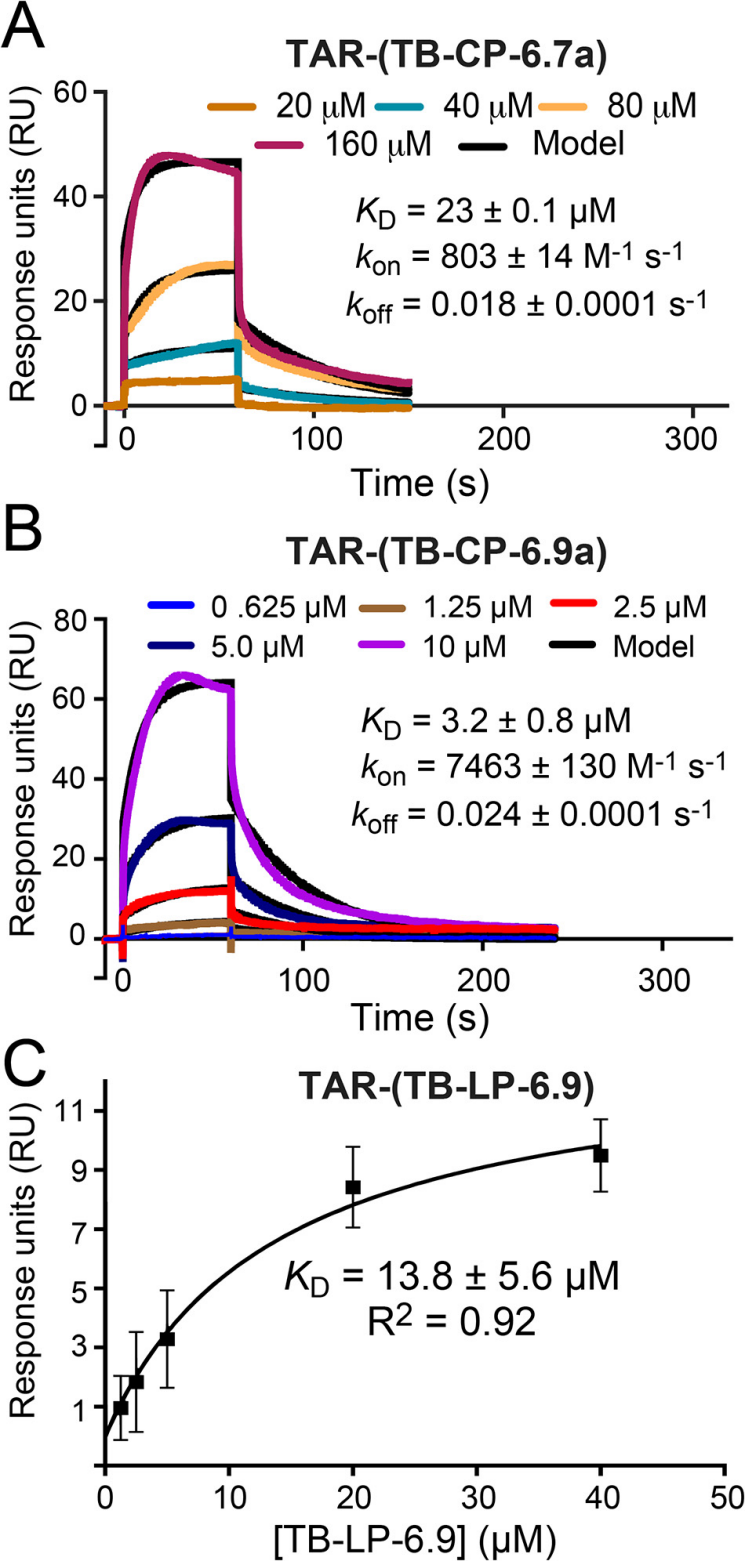
**Kinetic and equilibrium binding analysis of peptides TB-CP-6.7a, TB-CP-6.9a, and TB-LP-6.9 binding to HIV-1 TAR.***A*, representative sensorgrams from SPR showing cyclic peptide TB-CP-6.7a association with and dissociation from immobilized TAR RNA. Here and elsewhere, peptide concentrations are shown in the *key*; *colored lines* represent background-subtracted data; *black lines* indicate the global fit to a 1:1 binding model. The binding parameters obtained from the data set are shown; the χ^2^ (RU^2^) quality control metric for the fit was 0.76. For this and other experiments, average *k*_on_ and *k*_off_ rate constants and the apparent *K_D_* value from replicate runs are reported in Table 3. *B*, representative sensorgrams from SPR showing cyclic peptide TB-CP-6.9a association with and dissociation from immobilized TAR RNA. This experiment was conducted in the presence of a 100-fold molar excess tRNA. The χ^2^ (RU^2^) for the fit was 1.95. *C*, equilibrium binding analysis of linear peptide TB-LP-6.9 interacting with TAR; the average *K_D_* is shown.

Each peptide was then tested for TAR affinity using surface plasmon resonance (SPR). Importantly, cyclic peptide TB-CP-6.7a was found to interact with TAR with a *k*_on_ of 863.0 ± 59.0 m^−1^ s^−1^ and a *k*_off_ of 0.019 ± 0.0005 s^−1^ corresponding to an apparent *K_D_* of 22.0 ± 0.1 μm (Fig. 5*A* and Table 3). In contrast, linear peptide TB-LP-6.7 showed a very weak interaction toward TAR, and the SPR curves for this peptide could not be reliably fit using equilibrium analysis (data not shown). Nonetheless, these results demonstrated the feasibility of producing short cyclic peptide mimics of the isolated β2-β3 loop region from TBPs. In addition, these findings highlight the importance of a conformationally constrained backbone in TB-CP-6.7a to support the interaction with TAR.

**Table 3:** Average binding and kinetic parameters of TAR binding by TBP-derived peptides

Sample peptide	*k*_on_ × 10^2^	S.E. × 10^2^	*k*_off_ × 10^−2^	S.E. × 10^−2^	*K_D_*	S.E.	χ^2^	S.E.
	*m*^−*1*^*s*^−*1*^	*m*^−*1*^*s*^−*1*^	*s*^−*1*^	*s*^−*1*^	μ*m*	μ*m*	*RU^2^*	
TB-CP-6.7a	8.63	0.59	1.90	0.04	22.0	0.10	1.0	0.20
TB-CP-6.9a^a^	76.50	1.90	2.75	0.38	3.6	0.40	1.9	0.06
TB-LP-6.9	NA^b^	NA	NA	NA	13.8	5.60	NA	NA


a The binding assays leading to these measurements were conducted in the presence of 100-fold molar excess tRNA.

b NA, not applicable.

With the goal of obtaining a stronger binder for TAR, we applied the same strategy to generate cyclic peptide TB-CP-6.9a (Fig. S4C) based on the β2-β3 loop sequence of TBP6.9. We chose this variant because it contains four arginines and represents the tightest TAR binder in our ITC analysis (Table 2). A linear version of this peptide, TB-LP-6.9 (Fig. S4D), was also prepared. Interestingly, cyclic peptide TB-CP-6.9a was determined to bind TAR with a *k*_on_ of 7650 ± 190 m^−1^ s^−1^ and a *k*_off_ of 0.028 ± 0.004 s^−1^, corresponding to a *K_D_* of 3.6 ± 0.4 μm (Fig. 5*B* and Table 3). On the other hand, the linear peptide TB-LP-6.9 exhibited an ∼4-fold weaker binding affinity based on its apparent *K_D_* of 13.8 ± 5.6 μm, measured by use of equilibrium SPR (Fig. 5*C* and Table 3). Although the other peptides of this investigation showed poorer affinity in the presence of 100-fold molar excess tRNA, we observed that TB-CP-6.9a was capable of binding under this condition, demonstrating its specificity for TAR RNA.

The structures of these compounds in complex with TAR have yet to be determined. However, the TAR-binding capabilities of TB-CP-6.7a and TB-CP-6.9a—along with their higher affinity relative to their linear counterparts—suggest that these cyclic peptides mimic to a significant extent the co-crystal structures of their parental β2-β3 loops and their associated principles of arginine-mediated TAR recognition. At the same time, it is worth noting that the ∼6-fold difference in TAR-binding affinity of TB-CP-6.9a compared with TB-CP-6.7a is considerably larger than the ∼1.7-fold difference in TAR affinities measured for TBP6.9 *versus* TBP6.7 (Table 2). These results point to subtle yet important differences in the TAR recognition properties of the cyclic peptidomimetics compared with the corresponding TBPs.

### Cyclic peptide TB-CP-6.9a blocks Tat peptide binding to TAR

We next investigated whether cyclic peptide TB-CP-6.9a is able to block binding of the Tat ARM domain to TAR. Disrupting this interaction is challenging because the Tat ARM is known to span the entire length of the TAR major groove while making base-specific contacts and electrostatic interactions (19). Several studies have reported multiple ligand-binding sites within TAR that mainly target its bulge and major groove (43, 44, 45, 46). Accordingly, effective TAR binders must compete with the Tat ARM to disrupt a lengthy and robust network of RNA-protein interactions.

**Figure 6:**
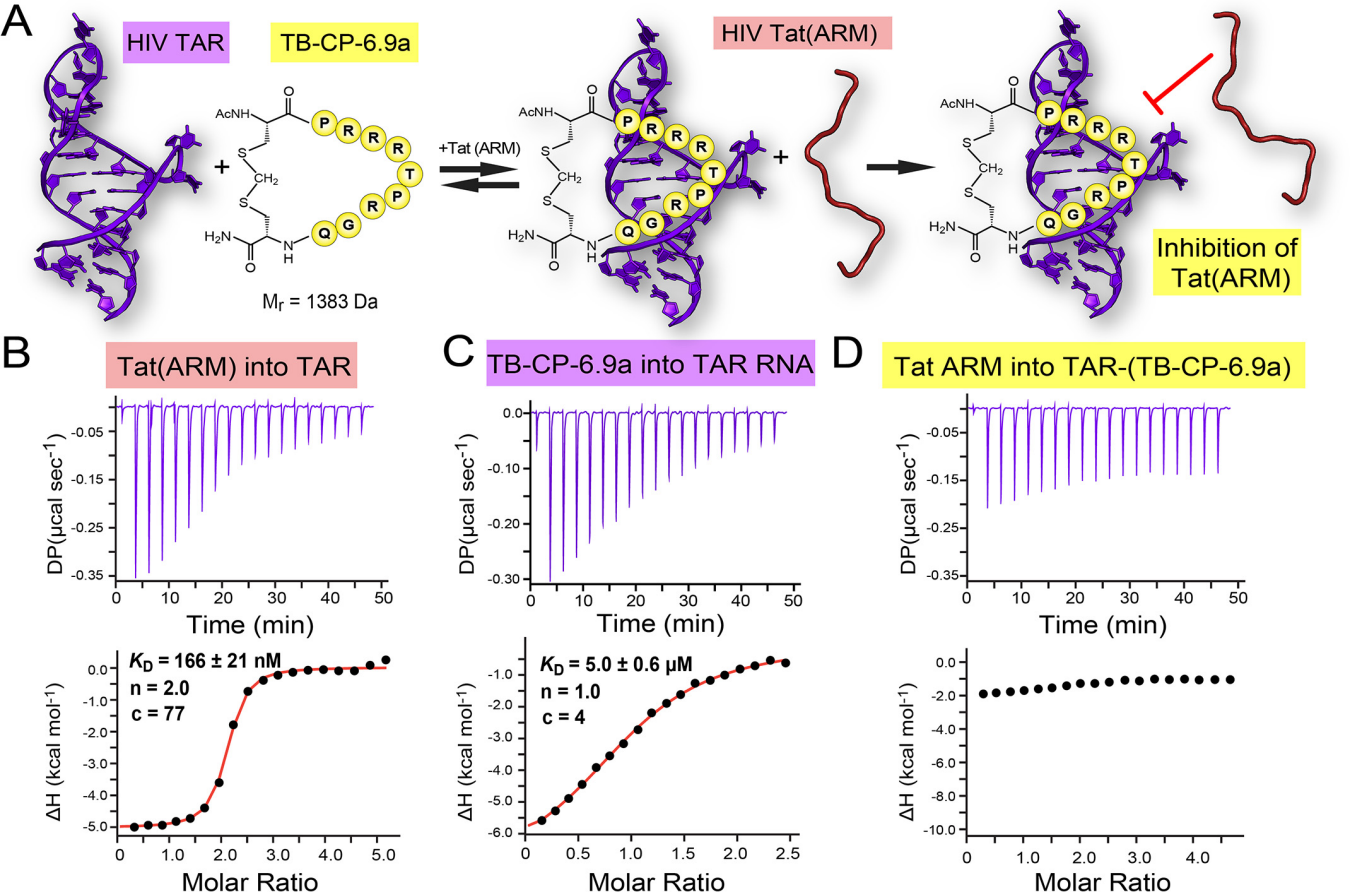
**Cyclic peptide competition with the Tat ARM for HIV TAR binding.***A*, schematic diagram of the TAR-Tat competition experiment wherein cyclic peptide TB-CP-6.9a is titrated initially into TAR RNA. The Tat ARM is titrated subsequently into the preformed complex. In a successful assay, the presence of TB-CP-6.9a competes with the Tat ARM to block the Tat-TAR interaction. The TAR structure used for this schematic was derived from the TAR-TBP6.9 complex, and the Tat ARM structure was derived from the lowest-energy conformation of the Tat-TAR complex (PDB 6MCE) (11). *B*, representative control titration of the Tat ARM peptide into HIV-1 TAR RNA produced an average *K_D_* of 135 ± 31 nm and with 2:1 stoichiometry, suggesting two high-affinity binding sites. For this and other experiments, the values indicated in *each ITC panel* correspond to individual thermograms, whereas average values are provided in Table 2. *C*, representative titration of cyclic peptide TB-CP-6.9a binding to TAR, which yielded an average *K_D_* of 5.3 ± 0.2 μm with 1:1 binding stoichiometry. *D*, representative competition titration in which the Tat ARM peptide was titrated into the product formed in *C*. The binding reaction shows no appreciable heats of binding. Each experiment in *B–D* was performed twice.

We used an ITC-based competition analysis derived from a previously reported assay (8) wherein a preformed TAR-(TB-CP-6.9a) complex was probed for peptide binding by the addition of the Tat ARM (Fig. 6*A*). As a control experiment, we first titrated the Tat ARM into TAR RNA. The interaction produced a strong heat of binding that gave a *K_D_* of 135.0 ± 31.0 nm (Fig. 6*B* and Table 2). Notably, the isotherm reveals a 2:1 Tat/TAR stoichiometry, implying the ability of the Tat ARM to bind at two sites. We then tested the inhibitory activity of cyclic peptide TB-CP-6.9a by performing two consecutive ITC experiments. First, we titrated TB-CP-6.9a into HIV-1 TAR RNA, which produced apparent 1:1 binding with an average *K_D_* of 5.3 ± 0.2 μm (Fig. 6*C* and Table 2). This value agrees well with the apparent *K_D_* of 3.6 ± 0.4 μm obtained by SPR (Table 3). We next titrated the Tat ARM into the preformed TAR-(TB-CP-6.9a) complex formed during ITC. We observed no significant heats of binding (Fig. 6*D*), demonstrating that TB-CP-6.9a formed a complex with TAR that occluded Tat-binding sites.

For comparison, similar experiments were carried out using linear peptide TB-LP-6.9. Consistent with its low binding affinity for TAR as determined by SPR (Fig. 5*C*), this peptide showed negligible heats of binding when titrated into TAR (Fig. S5A). Upon titration of the Tat ARM into the putative preformed TAR-(TB-LP-6.9) complex, an apparent two-phase binding curve was observed that yielded a *K_D_*_-1_ of 9.5 ± 2.1 nm and a *K_D_*_-2_ of 311 ± 7 nm (Fig. S5B). This experiment showed that whereas TB-LP-6.9 appears to alter Tat ARM peptide binding to TAR, it is unable to block the TAR-Tat ARM interaction. Altogether, these results suggest that the linear peptide cannot adopt a conformation that significantly blocks the sites of Tat binding within TAR, in contrast to its cyclic peptide counterpart.

## Discussion

HIV/AIDS is a pernicious public health threat with no vaccine or cure that has exacerbated other medical emergencies (47). As viral eradication efforts continue, an additional priority is to develop new antivirals that reduce morbidity and mortality while attaining a functional cure (48, 49). Disruption of the TAR RNA interaction with Tat represents a promising target to lock the virus into a latent state by blocking formation of a complex that is essential for completion of the viral life cycle (Fig. 1*A*) (50). On the pathway toward this goal, our structural and binding analyses of various lab-evolved TBPs revealed important principles of TAR molecular recognition by various β2-β3 loop sequences. Significant observations included the following: (i) no more than three β2-β3 loop arginines were operative simultaneously in recognition of the Hoogsteen edges of conserved guanine nucleobases at positions 26, 28, and 36; (ii) when positions 48 and 50 of β2-β3 loops were occupied by amino acids that promoted phosphate backbone binding or intrapeptide hydrogen bonding, TAR affinity was relatively high (*e.g.* TBP6.6, TBP6.7, and TBP6.9); (iii) a bulky aromatic group at position 48 was not well-tolerated (*e.g.* TBP6.25); and (iv) Arg-50 displaced Arg-52, causing suboptimal Gua36 recognition (*e.g.* TBP6.3 and TBP6.7 Q48R/T50R). Collectively, these observations illustrate why lab-evolved β2-β3 loops obey the known consensus sequence (Fig. 1*B*) while providing a rationale for the use of specific sequences in the synthesis of small cyclic peptides that target TAR.

**Figure 7:**
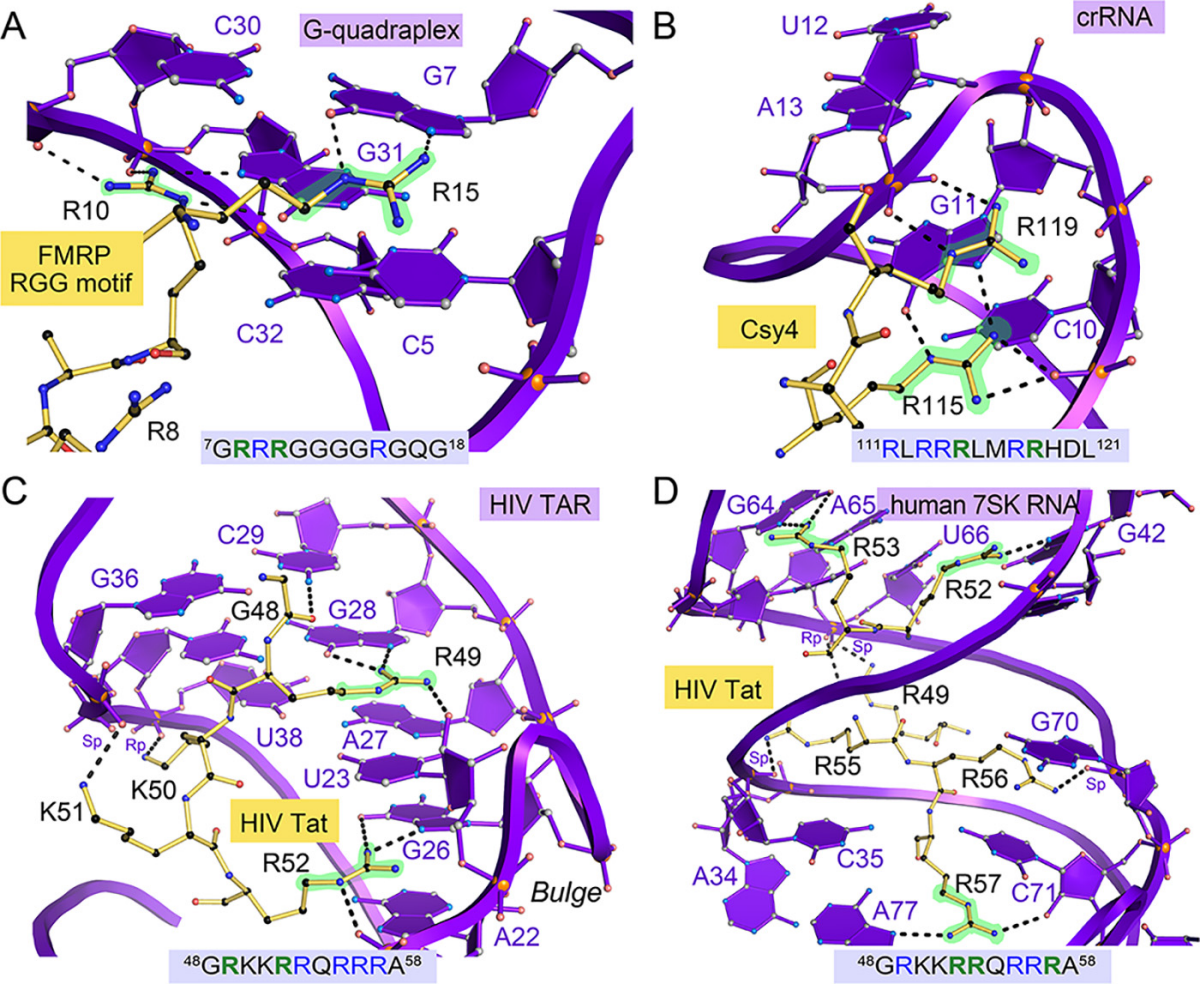
***Close-up views* depicting how various proteins containing ARMs use only a subset of arginines to recognize their cognate RNAs.***A*, *close-up view* of the FMRP-RGG-RNA interface (PDB entry 5DEA) (51) (*boxed area* in Fig. S6A). The β-hairpin RGG motif contains four arginines (sequence shown), but only two make base-specific contacts (*highlighted green*). Arg-10 and Arg-15 recognize the Hoogsteen edges of Gua31 and Gua7, but Arg-8 and Arg-9 do not make base-specific interactions. *B*, *close-up view* of the Csy4 endoribonuclease in complex with crRNA (PDB entry 4AL5) (52) (*boxed area* in Fig. S6B). The α-helical motif harbors six arginines, but only two participate in binding. Arg-115 reads the Hoogsteen edge of Gua11, and Arg-119 makes phosphate backbone interactions. Other arginines are either involved in salt-bridge interactions with the RNA backbone or do not make RNA contacts. *C*, *close-up view* of the HIV TAR-Tat interface (PDB entry 6MCE) (19) (*boxed area* in Fig. S6C). The ARM contains nine arginines, but only two make base-specific interactions. Arg-49 hydrogen-bonds to the Hoogsteen edge of Gua28. Similarly, Arg-52 hydrogen-bonds with the Hoogsteen edge of Gua26. Other arginines do not make base-specific interactions. *D*, *close-up view* of the HIV Tat-7SK RNA interface (PDB entry 6MCF) (11) (*boxed area* in Fig. S6D). The Tat ARM with nine arginines utilizes only three to make base-specific interactions. Specifically, Arg-52, Arg-53, and Arg-57 recognize the Hoogsteen edges of Gua42, Gua64, and Ade77. Arg-49, Arg-55, and Arg-56 do not make base-specific interactions.

A significant discovery from our co-crystal structures was that only three arginines from each β2-β3 loop could be used simultaneously for guanine recognition (Figs. 2 (*B* and *C*), 3 (*D–I*), and 4 (*B* and *C*)). Given this observation, we sought to identify whether other RNA-binding proteins showed similar limitations. A brief survey of relevant RNA-protein interactions featuring major-groove recognition by ARM elements revealed a parallel trend in arginine-mediated interactions. To illustrate, we considered the fragile-X mental retardation protein (FMRP) bound to G-quadraplex RNA (51). Here, the ARM of FMRP resides in a β-hairpin element harboring four arginines—analogous to TBP6.3, TBP6.9, and TBP6.7 Q48R/T50R. However, only two of the guanidinium moieties of the FMRP ARM make base-specific contacts (Fig. 7*A* and Fig. S6A). Arg-10 reads the Hoogsteen edge and phosphate backbone of Gua31, whereas Arg-15 interacts with the Hoogsteen edge of Gua7 (Fig. 7*A*). In another compelling example, the Csy4-crRNA complex reveals that the Csy4 endoribonuclease domain contains an α-helical arginine-rich motif that uses only one arginine in a cluster of six to make base-specific interactions (52) (Fig. 7*B* and Fig. S6B). Arg-115 reads the major-groove edge of Gua11 while salt-bridging with the phosphate backbone of Cyt10. In contrast, other arginines, such as Arg-119, contact the phosphate backbone (Fig. 7*B*) or make no RNA contacts whatsoever.

Use of distinct islands of arginines within a single ARM to recognize different RNA targets is exemplified by recent solution structures of HIV Tat in complex with human 7SK RNA and HIV TAR RNA (19). The Tat ARM contains six arginines separated by lysine and glutamine residues. Tat recognition of TAR uses only two of these arginines, Arg-49 and Arg-52, which bind major-groove guanine nucleobases (Fig. 7C and Fig. S6C). In contrast, the Tat ARM employs different arginines at Arg-53, Arg-56, and Arg-57 to recognize 7SK RNA in a base-specific manner (Fig. 7*D* and Fig. S6D) (19). These amino acid interactions are comparable with those of Arg-47, Arg-49, and Arg-52 in TBP6.6, TBP6.7, and TBP6.9 (Figs. 2 (*B* and *C*) and 3 (*E*, *F*, *H*, and *I*). Additionally, Lys-50 and Lys-51 of Tat make phosphate-specific backbone interactions with TAR, whereas Arg-55 interacts with the backbone of 7SK. Such contacts are similar to Arg-52 of TBP6.3 and Arg-48 of TBP6.9 (Figs. 3, *D*, *E*, *G*, and *H*).

Recognition of different RNAs by a single ARM that adopts different conformations has been dubbed “chameleonism” by Frankel (53). The HIV Tat-ARM exemplifies this concept because it uses an extended conformation during TAR recognition but assumes a hairpin for 7SK binding (19). The Jembrana disease virus Tat-ARM likewise binds as a β-hairpin to BIV-TAR but shows an extended conformation when bound to HIV-TAR (53). Finally, the HIV REV ARM assumes an α-helical conformation for RRE recognition (54) but forms an extended fold to recognize an anti-REV aptamer (55).

In contrast, Ellington and co-workers (56) subjected various ARM sequences to mutagenesis and observed that distinct subsets of arginines recognized different anti-ARM aptamers. Hence, aptamers could engage in cross-recognition of different ARM peptides. This finding parallels our observations for TAR binding to TBP6.3 and TBP6.7 Q48R/T50R, wherein Arg-50 displaced Arg-52 to read the Hoogsteen edge of Gua36, demonstrating sequence-dependent malleability of the β2-β3 loop (Figs. 3 (*D* and *G*) and 4 (*B* and *C*)). Comparable plasticity has been reported elsewhere for various arginine-rich motifs (54, 55, 56, 57, 58). At present, ample structural and biochemical evidence support the notion that only a handful of arginines are engaged in ARM recognition of a specific RNA target at any given time. These observations are well-aligned with our findings for the TBPs of this investigation.

To better understand the structural features needed for TAR targeting by short peptides, it is also instructive to compare the TAR-binding features of TBPs herein with those of JB181. The latter β-hairpin peptide was derived from BIV-TAR and has undergone multiple cycles of optimization to yield ultrahigh affinity for TAR (*K_D_* of 28.4 ± 4 pm) and *bona fide* antiviral activity in cell culture (25). Unlike TBPs, the innovative JB181 peptide uses branched-chain amino acids between base-recognition arginines to promote hydrophobic core packing of the hairpin β-strands (Fig. S7A). The arginine content in this family of head-to-tail cyclic peptides was varied from 5 to 7 to elicit high affinity (23, 24, 35, 39). Like TBP6.9, JB181 contains four arginines, but the latter peptide also uses additional positively charged unnatural amino acids to favor binding at the TAR bulge (25). NMR data confirm that the Arg-3 and Arg-5 guanidinium groups of JB181 make Hoogsteen-specific contacts to Gua26 and Gua28 in TAR (Fig. S7B)—interactions that parallel those of Arg-47 and Arg-49 in all TBPs of this investigation (Figs. 2 (*B* and *C*) and 3 (*D–I*)). Unlike the TBPs, however, JB181 does not use amino acid side chains to engage in intrapeptide stabilization, such as Thr-48 and Thr-50 of TBP6.6 (Fig. 3 (*F* and *I*) and Fig. S3A). Instead, the backbone of JB181 participates in multiple carbonyl oxygen to amide hydrogen bonds that stabilize the β-hairpin fold, likely predisposing the peptide conformation to recognize RNA. Although, Arg-8 and Arg-9 of JB181 did not make any appreciable interactions with TAR, Dab1 (2,4-diaminobutyric acid) and Lys-6 bound to the phosphate backbone and O4 of Uri25 in TAR. Such interactions are strikingly analogous to Arg-48 and Arg-52 in the context of TBP6.7 Q48R/T50R (Fig. 4, *B* and *C*).

Perhaps significantly, one interaction that was not captured by JB181 or its precursors was an “arginine-fork” interaction (28) between Arg-47 and conserved Gua26. Indeed, we observed this interaction in all TBP-TAR complexes, wherein the Nη1 and Nη2 amino moieties of the Arg-47 guanidinium group hydrogen bond to O6 and N7 of Gua26 (Figs. 2*B* and 3 (*D–F*)). Simultaneously, Nε and Nη2 of Arg-47 hydrogen-bond to the pro-*R*_p_ and O5′ oxygens within the Uri23 phosphate backbone, thus giving a two-pronged “fork” readout; the Arg-47 guanidinium is flanked by cation-π contacts to Ade22 and Uri23 that further strengthen the mode of binding. The recent HIV-1 Tat-TAR complex reveals an analogous interaction between Arg-52 and Gua26 (Fig. 7*C*). Significantly, methylation analysis and mutagenesis of each arginine in the arginine-rich motif of HIV Tat revealed that Arg-52 alone is sufficient for TAR binding and *trans*-activation (59, 60, 61, 62). We hypothesize that our peptide, TB-CP-6.9a, maintains the critical Arg-to-Gua26 interaction seen in TBPs, which effectively competes with Tat-ARM binding. Our ITC analysis is consistent with the idea that TB-CP-6.9a contains a binding site that overlaps with Tat. Efforts to determine co-crystal structures of our cyclic peptides in complex with TAR are under way.

Although JB181 was generated by structure-based design, our investigation herein evaluated the feasibility of generating small TAR-binding cyclic peptides derived from the lab-evolved β2-β3 loops of TBPs. Previously, we reported a 20-mer cyclic peptide, peptide 1 (2,577 Da) that comprises the entire β2-β3 strand-loop-strand element of TBP6.7 cyclized via a perfluoroaryl linker (28). Peptide 1 was determined to bind TAR with a *K_D_* of 1.8 ± 0.5 μm based on a fluorescence assay. Here, we report the successful development of a significantly smaller (11-mer; 1,383 Da) cyclic peptide, TB-CP-6.9a, that is able to interact with TAR with a comparable *K_D_* of 3.6 ± 0.4 μm (Table 3). This result demonstrates that the TAR-binding properties of the isolated β-hairpin motif in TBP can be largely reproduced using a cyclic peptide scaffold that spans only the β2-β3 loop region. In addition, TB-CP-6.9a was shown to effectively block the interaction of TAR with the Tat ARM (Fig. 6), thus representing a viable new inhibitor of this RNA-protein interaction. Notably, the linear peptide counterpart, TB-LP-6.9, not only binds TAR with weaker affinity but it is also unable to block the interaction of TAR with Tat ARM (Fig. S5). This result highlights the importance of the cyclic structure of TB-CP-6.9a in enabling the β2-β3 loop sequence to adopt a relevant conformation for efficient interaction with TAR at a binding site that overlaps that of Tat. The behavior of the linear peptides can be attributed to the underlying chameleonism (*i.e.* conformational adaptability) of their arginine-rich sequences, which likely undermines their ability to recognize the target RNA with high affinity and/or specificity.

Interestingly, cyclic peptide TB-CP-6.9a binds TAR with 6-fold higher affinity than TB-CP-6.7a, which contrasts with the 1.6-fold difference in affinity for the corresponding loop sequences when embedded within their TBP scaffolds (Table 2). The affinity improvement of TB-CP-6.9a *versus* TB-CP-6.7a is mainly driven by an increase in association rate constant (*k*_on_) for the interaction with TAR (8-fold increase; Table 3). Based on these results, we hypothesize that the improved performance of TB-CP-6.9a may derive from its ability to more readily adopt a productive conformation for interaction with TAR compared with TB-CP-6.7a. Indeed, similar *k*_on_-driven affinity improvements have been reported for other cyclic peptides whose bioactive conformation was stabilized by backbone modifications (63). These results also suggest that variation of the peptide sequence and intramolecular linkage could provide a means to further improve the TAR-binding properties of these compounds, which will be the object of future studies.

Overall, by determining the crystal structures of four new HIV TAR-TBP complexes, we generated new insights into the molecular recognition principles used by a family of lab-evolved proteins to readout conserved major-groove guanines of TAR. Although it was hypothesized originally that each TBP variant would recognize TAR using fundamentally different modes of binding (8), we demonstrated here that each TBP binds TAR using a conserved subset of arginine-to-guanine interactions within a lab-evolved β2-β3 loop. Each loop adopted a highly similar conformation (Fig. 2, *D* and *E*). Such knowledge was instrumental for the development of a small cyclic peptide mimic of the β2-β3 loop that is able to bind TAR and inhibit its interaction with the Tat ARM. Whereas further optimization of this compound is warranted, this work paves the way to the development of a new class of inhibitors of the TAR-Tat interaction. It also demonstrates the feasibility of using compact cyclic peptides to target viral RNAs—a subject of great concern given the ongoing coronavirus pandemic (64, 65).

## Experimental procedures

### Expression and purification of TBPs

TBP6.3, TBP6.6, TBP6.7, TBP6.7 Q48R/T50R, TBP6.6, and TBP6.25 DNA were each prepared from a synthetic gene cloned into pUC57 (GeneScript Inc.). Variants were derived from the human U1A protein sequence modified to produce sequences generated from yeast display (28, 66) (Fig. 1*B*); in addition, the Y31H/Q36R mutant was integrated into each variant to promote crystallization (28, 66). TBP inserts were excised by restriction digests using enzymes that cut at unique NcoI and BamHI sites (NEB Inc.). Inserts were gel-purified, ligated into restriction-digested pET28a treated with alkaline phosphatase, and transformed into DH5α cells. Plasmids were isolated and verified by DNA sequencing (GeneWiz Inc.). For protein expression, plasmids were transformed into *Escherichia coli* BL21(DE3) (NEB Inc.). Freshly streaked colonies grown on Luria–Bertani (LB) agar plates under selection conditions at 37 °C were used to inoculate overnight LB liquid cultures containing 50 μg/ml kanamycin. The overnight cultures were used to start large-scale LB growths at 37 °C that were induced during mid-log phase by 0.5 mm isopropyl 1-thio-β-d-galactopyranoside added to the media. Cells were harvested after 4 h of growth at 20 °C, and pellets were frozen in liquid N_2_. Cells were thawed in a cell lysis buffer (CLB) containing 0.05 m Na-HEPES, pH 7.5, 0.5 m NaCl, 0.02 m imidazole, pH 8.0, 0.0005 m EDTA, 0.005 m β-mercaptoethanol (β-ME) and 0.01% (v/v) Brij35; the cell slurry was made 2 mg ml^−1^ in lysozyme (VWR). After 20 min, cells were sonicated, and the clarified supernatant was applied in batch to nickel-nitrilotriacetic acid resin (Pierce) equilibrated with CLB. After 2 h of nutation at 4 °C, resin was poured into a 1.5 × 10-cm gravity-flow column (CrystalCruz), washed with 40 column volumes of CLB and 2 column volumes of wash buffer containing 0.05 m Na-HEPES, pH 7.0, 0.3 m NaCl, 0.04 m imidazole, pH 7.5, 0.005 m EDTA, 0.005 m β-ME, and 0.01% (v/v) Brij35. Elution was in 3-ml fractions using an elution buffer containing 0.05 m Na-HEPES, pH 7.0, 0.15 m NaCl, 0.2 m imidazole, pH 7.5, 0.005 m EDTA, 0.005 m β-ME, and 0.01% (v/v) Brij35. Fractions were pooled based on absorption at 280 nm and diluted with wash buffer to a final imidazole concentration <0.02 m. TEV (67) was added (1:100 TEV/TBP), and the mixture was incubated at 4 °C to remove the His_6_ tag. After 16 h, the reaction was incubated in batch with pre-equilibrated nickel-nitrilotriacetic acid, and the supernatant was collected. The protein was loaded at 0.5 ml min^−1^ onto a 5-ml HiTrap SP FF column (GE) using an ÄKTA Pure (GE Lifesciences). The sample was washed and eluted using a linear gradient comprising 0.15–0.85 m NaCl, 0.05 m Na-HEPES, pH 7.0, 0.0025 m EDTA, and 0.00025 m β-ME. TBP6.3 and TBP6.9 were purified similarly except that a 0.15–1.5 m NaCl salt gradient was used for the HiTrap SP FF column to reduce nonspecific RNA interactions. Each concentrated protein sample was gel-filtered using a HiPrep (16/60) Sephacryl S-300 HR column (Cytiva). TBPs (11.5 kDa) exhibited higher retention than predicted by mass, eluting at or >1 column volume. The yield was 2–3 mg/liter of cells.

### Crystallization and X-ray data collection

The HIV TAR 27-mer RNA (Fig. 2*C*) was generated by chemical synthesis (Horizon Discovery) and purified as described (28). The RNA was dissolved in 0.01 m Na-HEPES, pH 7.5, to a concentration of 0.4 mm and heated at 65 °C. After 3 min, the RNA was diluted 10-fold with folding buffer (0.01 m Na-HEPES, pH 7.5, 0.05 m NaCl, and 0.002 M MgCl_2_) and incubated at 65 °C for 2 min. The RNA was cooled overnight to room temperature. TBP was titrated dropwise with gentle vortexing into a microcentrifuge tube containing the folded RNA to achieve a 1.2:1 molar ratio (48 μm protein to an equal volume of 40 μm RNA). The mixture was incubated at room temperature for 0.5 h and concentrated using a Nanosep 3K Omega spin-filter (PALL) to 10–12 mg ml^−1^ based on absorption at 280 nm. The final complex was 0.2-μm-filtered (Millex, EMD) and subjected to hanging-drop broad screens using a Mosquito^TM^ robot (SPT Labtech). An equal volume of well solution was added to 0.2 μl of TAR-TBP complex. Most crystals appeared in 4–7 days at 20 °C as thin plates with a half-octagon habit. Crystals were typically 0.15 × 0.07 × 0.04 mm in size. TAR-TBP6.7 Q48R/T50R crystals grew directly from the JCSG-Plus screen (Jena Bioscience) from a well solution comprising 20% (w/v) PEG3350 and 0.2 m (NH_4_)_2_NO_3_. TBP6.9-TAR crystals grew directly from a Natrix broad screen (Hampton Research) from a well solution of 17.5% (w/v) PEG4000, 0.005 m MgCl_2_·6H_2_O, and 0.05 m MES monohydrate, pH 6.0. Other crystals were optimized from broad screens and were prepared by hanging-drop vapor diffusion using VDX plates (Hampton Research). For TBP6.3, an equal volume of well solution comprising 17.5% (w/v) PEG3350, 0.1 m Na-cacodylate, pH 6.0, and 0.2 m NH_4_HCO_2_ was added to 2 μl of TAR-TBP6.3 complex with equilibration over 1 ml of mother liquor at 20 °C. TAR-TBP6.6 crystals grew from 17% (w/v) PEG5000 MME, 0.1 m NaCl, 0.002 m (NH_4_)_2_SO_4_, and 0.05 m Na-cacodylate, pH 7.0. Cryo-protection of all crystals was by serial transfer of crystals into mother liquors supplemented in five steps from 5 to 20% (v/v) glycerol followed by snap cooling in liquid N_2_. X-ray data were recorded at the Stanford Synchrotron Radiation Lightsource. Data reduction statistics are shown in Table 1.

### Structure determination and refinement

X-ray diffraction data were reduced using the SSRL autoxds script (https://smb.slac.stanford.edu/facilities/software/xds/#autoxds_script), which employed the XDS and CCP4 packages (68, 69). All four structures were determined by molecular replacement using PHASER as implemented in PHENIX using the TAR-TBP6.7 structure as a search model (28, 70, 71). The structures were manually rebuilt using COOT and refined using PHENIX (71, 72). Refinement statistics are provided in Table 1. All cartoons and schematic diagrams derived from coordinates were produced in PyMOL (Schrödinger, LLC). Least-squares superpositions were performed in CCP4 using Lsqkab (69, 73).

### Isothermal titration calorimetry

The HIV-1 TAR 27-mer was produced by *in vitro* transcription as described (74). The transcribed RNA was purified by denaturing gel electrophoresis, desalted, and lyophilized. RNA was dissolved in 0.01 m Na-HEPES, pH 7.5, and heated at 65 °C. After 3 min, 65 °C ITC buffer (0.05 m Na-HEPES, pH 7.5, 0.05 m NaCl, 0.05 m KCl, 0.002 m MgCl_2_, and 0.002 m β-ME) was pipetted into the RNA, followed by 2 min of incubation at 65 °C; the sample was cooled overnight to room temperature. Each sample was dialyzed overnight at 4 °C against 4 liters of ITC buffer. Following co-dialysis with RNA, protein samples were diluted in dialysis buffer to concentrations ∼10-fold higher than RNA. ITC measurements were conducted using a PEAQ-ITC (Malvern Panalytical) with protein in the syringe and RNA in the cell (75, 76, 77). Experiments were conducted at 20 °C unless noted. The time between injections was 150 s with a total of 19 injections. Thermograms were analyzed with PEAQ-ITC Analysis software using a 1:1 binding model. Average thermodynamic parameters are provided in Table 2. Experiments were repeated in duplicate.

### Macrocyclic peptide synthesis

Solid-phase peptide synthesis was carried in syringes equipped with Teflon filters (Torviq). Analytical HPLC was performed on a Shimadzu (LC-2010A HT) using an analytical C18 column (Hypersil GOLD, 4.5 × 250 mm) at a flow rate of 1 ml/min. Semipreparative HPLC was performed on an Agilent 1200 using a Hypersil GOLD C18 semipreparative column (10 × 250 mm) at a flow rate of 2.5 ml/min^−1^. Commercial reagents were used without further purification. Resin was purchased from Creosalus. Protected amino acids, activating reagents, and HATU were from Chem-Impex, Int. The buffer compositions included Buffer A, comprising 0.1% TFA in water, and Buffer B, comprising 0.1% TFA in acetonitrile.

The synthesis was carried out using Fmoc–solid-phase peptide synthesis on Knorr amide resin (0.4 mmol/g on a 0.1-mmol scale). Peptide synthesis was performed manually in syringes in the presence of 0.4 mmol amino acid (4 eq), 0.4 mmol HATU (4 eq), and 0.8 mmol DIPEA (8 eq). Initially, preswollen resin was treated with 20% piperidine in DMF containing 50 mmol of hydroxybenzotriazole (3 cycles 3-5-3 min) to remove the Fmoc-protecting group. Amino acids were coupled using the aforementioned reagent conditions. After coupling of the final amino acid, the Fmoc group was removed. The free amine at the N terminus was acetylated using 10 eq of acetic anhydride and 20 eq of DIPEA in DMF.

The polypeptide product was cleaved from resin as follows: the resin was washed with DMF-DCM and dried by vacuum; the product was cleaved using a mixture of TFA/triisopropylsilane/water (95:2.5:2.5) for 2 h at room temperature; the cleavage mixture was filtered, and the combined filtrate was added dropwise to a 10-fold volume of cold diethyl ether and centrifuged; the supernatant was removed, and the precipitated crude peptide was dissolved in 10 ml of acetonitrile/water (1:1) and lyophilized.

Peptide cyclization was performed as follows: the lyophilized peptide (4 mmol) was dissolved in 20 ml of 0.05 m ammonium bicarbonate buffer in 50% acetonitrile, 50% acetonitrile/ammonium bicarbonate buffer, pH 8.5. Tris(2-carboxyethyl)phosphine (TCEP) (8 mmol, 2 eq) was added to the peptide solution and incubated at 37 °C for 1 h. The diiodo-methane linker (24 mmol, 6 eq) was dissolved in DMF and added to the peptide solution and allowed to incubate at 37 °C for another 12 h. Progress of the reaction was monitored by MALDI-MS.

After cyclization, the solution was lyophilized. The cyclic peptide was purified by semi-preparative HPLC using a gradient of 20–75% Buffer B in 30 min, which resulted an average overall yield of 10–12%. Representative MS data and HPLC traces for each peptide are provided in Fig. S4.

### Surface plasmon resonance

Biotinylated-TAR 27-mer was deprotected and desalted by the manufacturer (Horizon Discovery). The RNA was folded as described for ITC (above). The RNA was immobilized on CM5 chips (Cytiva) conjugated to Neutravidin using an SPR buffer comprising 0.010 m HEPES, pH 7.5, and 0.15 m NaCl. A 200 nm stock of biotinylated TAR dissolved in SPR buffer was flowed over the chip surface to achieve 1200 RU. Analysis of TB-CP-6.7a proceeded in SPR buffer. To reduce nonspecific binding during TB-CP-6.9a assays, a 100-fold molar excess of yeast tRNA was added to SPR buffer. The flow rate for kinetics experiments was 75 μl/min. Cyclic peptides at various concentrations (10–160 μm for TB-CP-6.7a and 0.625–20 μm for TB-CP-6.9a) were injected for 60 s and allowed to dissociate for 180 s. To regenerate the RNA, 2 m NaCl was injected for 60 s. Experimental data were processed using the double-referencing method (78). The buffer-subtracted sensorgrams were fit to a 1:1 binding model using Biacore T200 analysis software to determine rate constants (*k*_on_ and *k*_off_) and the apparent equilibrium binding constant (*K_D_*) (79). The results were plotted using Prism software (GraphPad Inc.). The *K_D_* for the equilibrium binding measurements on TB-LP-6.9 was determined by taking the average response from a 5-s window at equilibrium (*R*_eq_) for each peptide injection and plotting *versus* the peptide concentration using Prism software; data were then fit to a one-site binding model. The kinetic and equilibrium experiments were repeated in duplicate.

### ITC of peptides and competition assays

For competition experiments, TAR RNA was folded as described (above) and dialyzed overnight against 4 liters of ITC buffer (0.05 m Na-HEPES, pH 7.5, 0.05 m NaCl, 0.05 m KCl, and 0.002 m β-ME). The Tat ARM sequence (19) was GISYGRKKRRQRRRAHQ, which included an acetylated N terminus and an amidated C terminus. The peptide was HPLC-purified by the manufacturer (Genscript, Inc.). For Tat-TAR titration, a 150 μm stock of the Tat ARM was titrated into 10 μm TAR RNA in the ITC sample cell. Resulting isotherms were best fit to a one-site binding model with a 2:1 stoichiometry. Comparable ITC binding experiments in which 400 μm peptide TB-LP-6.9 was titrated into 20 μm TAR revealed no appreciable heats of binding (Fig. S5A). For the TB-CP-6.9a competition assay with the Tat ARM, 400 μm cyclic peptide was titrated into 20 μm TAR in the sample cell, and the resulting isotherm fit best to a one-site model. Next, the Tat ARM peptide at a concentration of 500 μm was titrated into the preformed TAR-(TB-LP-6.9) complex retained in the sample cell from the prior experiment. For the competition assay with linear peptide, 500 μm TB-LP-6.9 was titrated into 20 μm TAR in a dropwise manner and incubated on the bench at room temperature for 1 h. The Tat ARM at a concentration of 400 μm was then titrated into the preformed complex in the sample cell. This titration was best fit to a two-site binding model. All curves were fit using MicroCal PEAQ-ITC Analysis software (Malvern Panalytical). Average thermodynamic parameters are provided in Table 2. Each titration and competition assay was repeated in duplicate.

## Data availability

Coordinates and structure factor amplitudes have been deposited into the Protein Data Bank as entries 6XH0, 6XH1, 6XH2, and 6XH3.

## Author Profile

Sai Shashank Chavali
