# Supplementary material for: Co-crystal structures of HIV TAR RNA bound to lab-evolved proteins show key roles for arginine relevant to the design of cyclic peptide TAR inhibitors
Source: J Biol Chem. 2021 Jan 13;295(49):16470–86. doi: 10.1074/jbc.RA120.015444 (PMC7864049; doi:10.1074/jbc.RA120.015444)
Supplement: Supplementary file 1 [file mmc1.pdf]

Supporting Information

Co-crystal structures of HIV TAR RNA bound to lab-evolved proteins show key roles for arginine relevant to the design of cyclic peptide TAR inhibitors

Sai Shashank Chavali[1], Sachitanand M. Mali[2], Jermaine L. Jenkins[1], Rudi Fasan[2] & Joseph E. Wedekind[1*]

[1]Department of Biochemistry & Biophysics and Center for RNA Biology, University of Rochester School of Medicine & Dentistry, Rochester NY 14642, USA.

[2]Department of Chemistry, University of Rochester, Rochester NY 14627, USA.

*Corresponding author: Joseph E. Wedekind

E-mail: joseph.wedekind@rochester.edu.

Running Title: *TAR recognition by arginine-rich cyclic peptides*

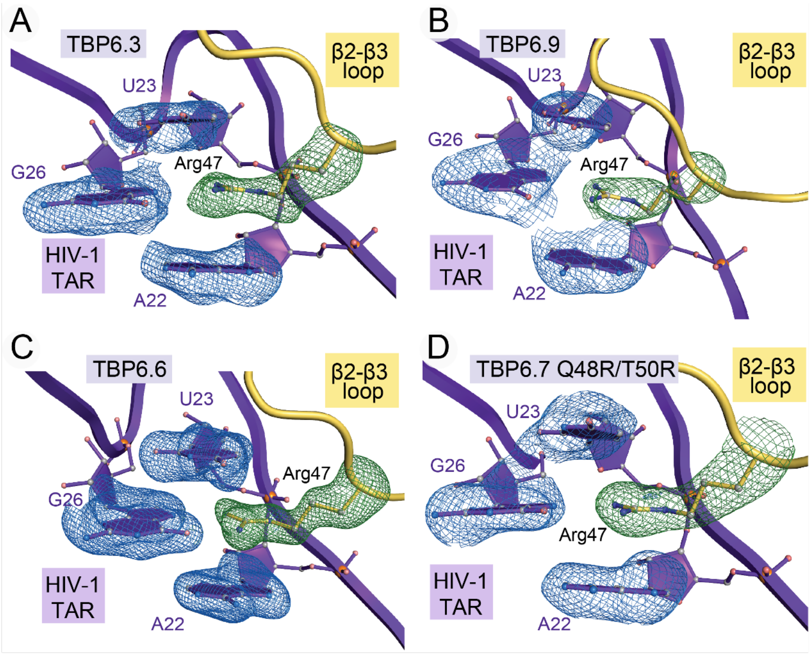

Figure S1 | Representative simulated-annealing-omit electron density maps for Arg47 of each TAR-TBP complex of this investigation. (**A**) A reduced-bias simulated-annealing-omit (mF_o-DF_c) electron density map (80,81) of the TAR-TBP6.3 co-crystal structure at 2.35 Å resolution calculated from phases of the final refined coordinates. Here and elsewhere atoms of the Arg47 sidechain and those of bases Ade22, Uri23 and Gua26 were excluded from the phase calculation. The map is contoured at the 3.5σ level and the final coordinates are depicted as ball-and-stick models. The density highlights the chemical details of the arginine-fork (28,60), wherein Arg47 is stacked between Uri23 and Ade22 while simultaneously recognizing the Hoogsteen edge of Gua26 and the phosphate backbone of Uri23. (**B**) A reduced-bias simulated-annealing-omit electron density map of the TAR-TBP6.9 co-crystal structure calculated at 3.10 Å resolution. The map is contoured at the 4.5σ level, which illustrates preservation of the arginine-fork conformation as in panel **A**. (**C**) A reduced-bias simulated-annealing-omit electron density map of the TAR-TBP6.6 co-crystal structure calculated at 1.71 Å resolution. The map is contoured at the 3.5σ level, which illustrates preservation of the arginine-fork conformation. (**D**) A reduced-bias simulated-annealing-omit electron density map of the TAR-TBP6.7 Q48R/T50R co-crystal structure calculated at 2.60 Å resolution. The map is contoured at the 3.5σ level, which illustrates preservation of the arginine-fork conformation.

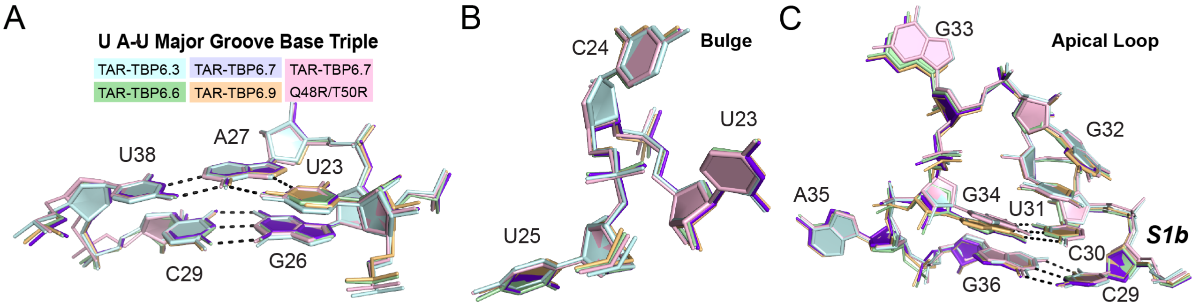

Figure S2 | Superposition of RNA structural elements derived from the co-crystal structures of TAR-TBP complexes in this investigation. (**A**) Close-up view of the superimposed Uri•Ade-Uri major-groove base triple comprising Uri23, Ade27 and Uri38. This tertiary interaction is a hallmark of the ligand-bound TAR conformation (12), which is preserved across all TAR-TBP co-crystal structures herein. The superimposed coordinates are the same as those shown in **Fig. 2D**. Here and elsewhere dashed lines indicate hydrogen bond interactions. (**B**) Close-up view of the superimposed central bulge that interrupts stem s1a and s1b. The bulge extrudes Cyt24 and Uri25 from the core and remains identical in all co-crystal structures of this investigation. (**C**) The apical hexaloop borders helix s1b at a canonical Watson-Crick closing base-pair between Gua36 and Cyt29, as observed in the TBP6.7-TAR structure (28). Gua34 and Cyt30 of the apical loop form a canonical pair that leads to bulged base Ade35. The loop conformation is unperturbed in all TAR-TBP structures herein.

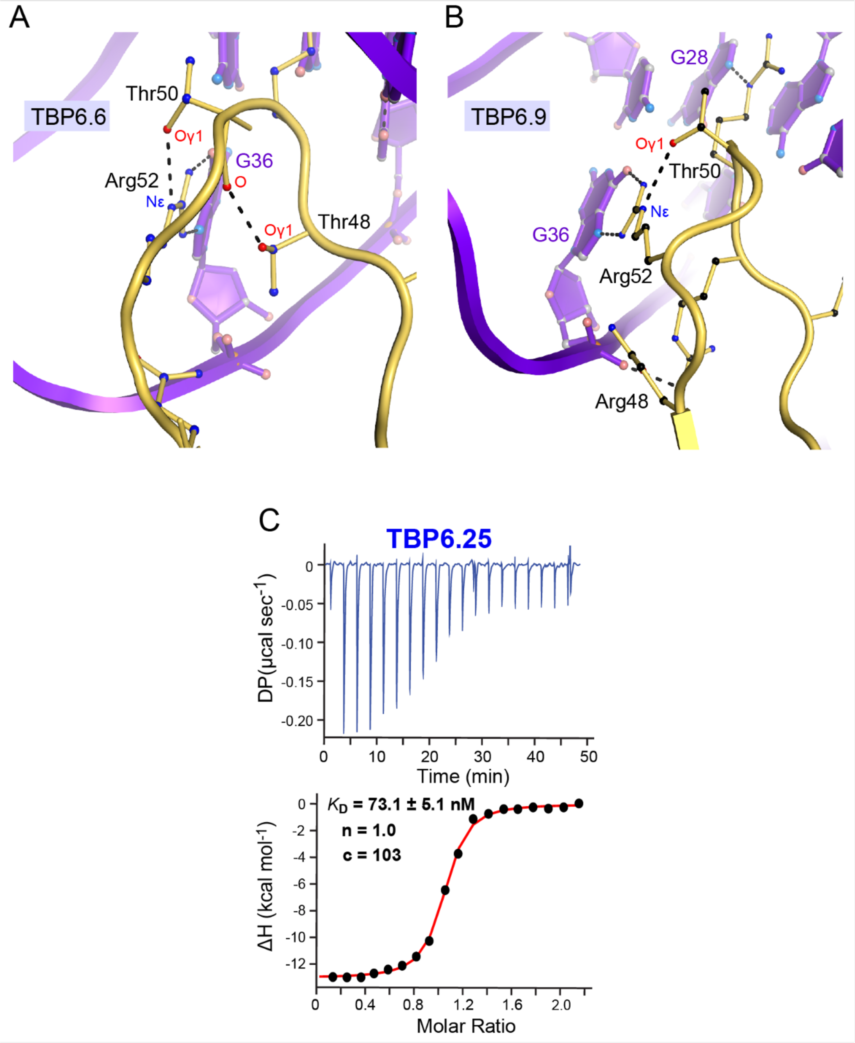

Figure S3 | Ball-and-stick diagrams of threonine-mediated stabilization of the β2-β3 loops from TBP6.6 and TBP6.9, and ITC analysis of TBP6.25. (**A**) Close-up view of intrapeptide hydrogen bond interactions observed in the TAR-TBP6.6 complex. The view shows Oγ1 of Thr48 interacting with the carbonyl oxygen of Thr50; also depicted is the interaction between Oγ1 of Thr50 and Nε of Arg52, which stabilizes the Arg52 rotamer for Gua36 recognition. (**B**) Close-up view of hydrogen bond interactions in the TAR-TBP6.9 complex; the view is similar to panel **A**. The drawing illustrates the preserved backbone interaction between Oγ1 of Thr50 to Nε of Arg52. In TBP6.9, threonine has been replaced by arginine at position 48, leading to a new salt bridge between the guanidinium and the phosphate group of Gua36. (**C**) Representative ITC thermogram resulting from titration of TBP6.25 into TAR. The average value from replicate analysis is provided in **Table 2**.

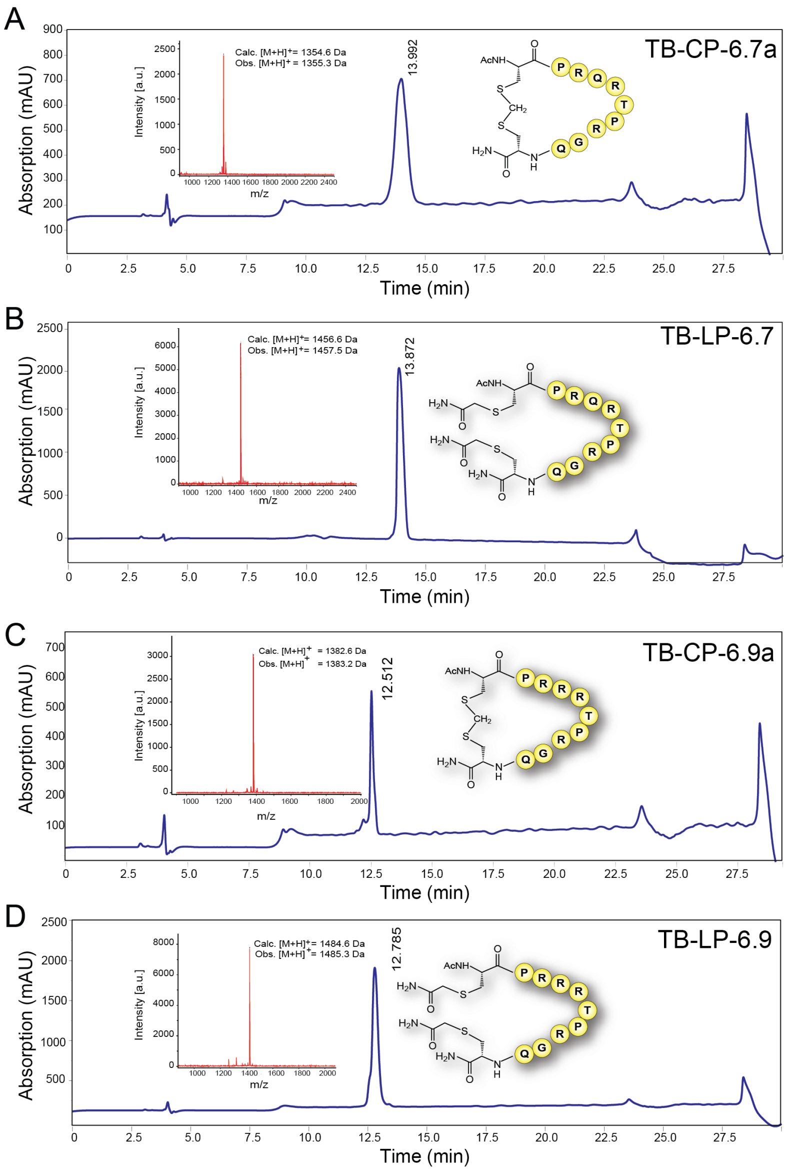

Figure S4 | Representative HPLC chromatograms and mass spectrometry analysis of TBP-derived β2-β3 loop peptides synthesized for this investigation. (**A**) HPLC profile of cyclic peptide TB-CP-6.7a using a C18 column and a gradient of 20-75% Buffer B (0.1% TFA acetonitrile) eluted over 30 min. (*Inset*) Mass spectrometry of the pure peptide. The sequence of the linear peptide is shown. (**B**) Analysis of linear peptide TB-LP-6.7 as described in panel **A**. Here and elsewhere, the free thiol resulting from Trt (Triphenylmethyl) removal during peptide cleavage from resin was modified with iodoacetamide to protect the cysteine. (**C**) Analysis of cyclic peptide TB-CP-6.9a as described in panel **A**. (**D**) Analysis of linear peptide TB-LP-6.9 as described in panel **A**.

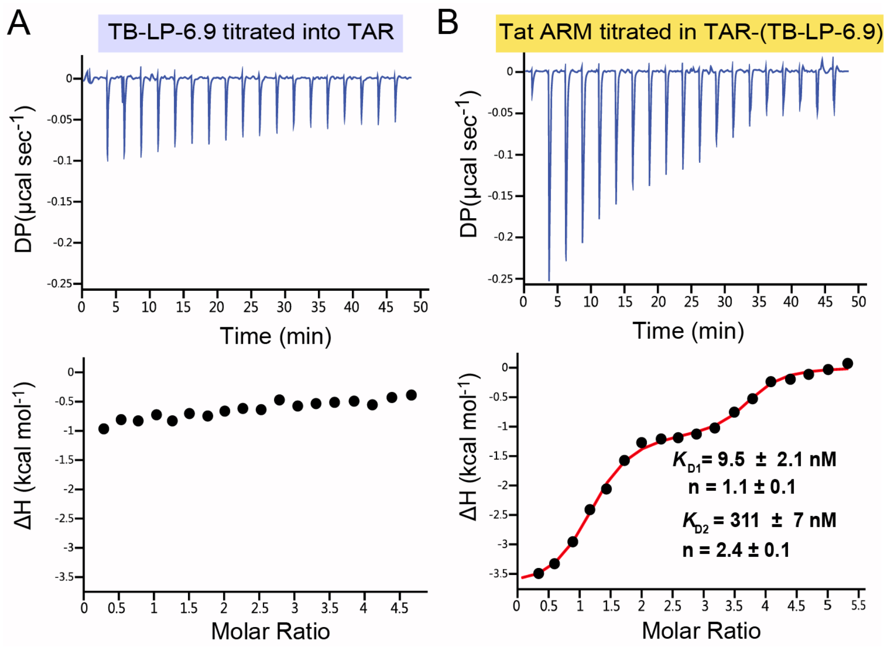

Figure S5 | Representative control experiment for the TAR-Tat binding competition assay using poor binder TB-LP-6.9. (**A**) Representative ITC analysis of linear peptide TB-LP-6.9 shows insufficient heats of binding to generate a reliable fit. (**B**) Representative ITC analysis of Tat titration into a pre-formed complex of TAR mixed with TB-LP-6.9. Tat exhibits non-identical two-site binding in the presence of a molar excess of TB-LP-6.9. This experiment demonstrates that Tat interacts strongly with TAR in the presence of a peptide that differs from TB-CP-6.9a (**Fig. 6**) only by cyclization.

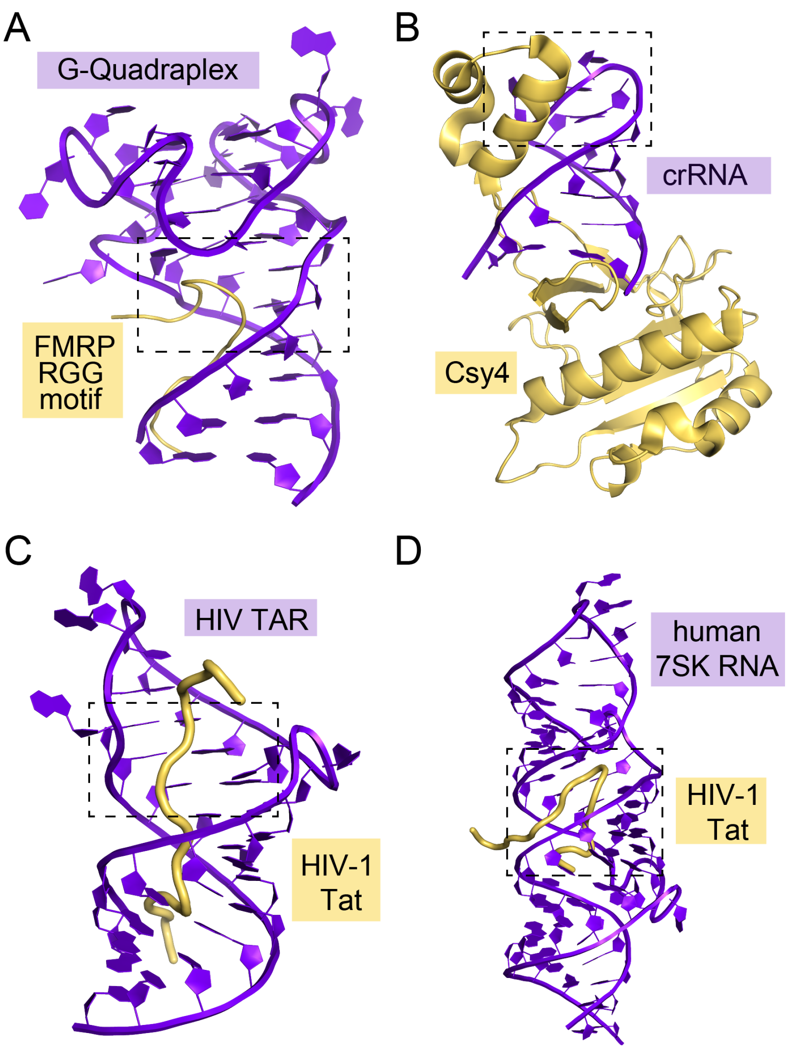

Figure S6 | Global views of naturally occurring ARM proteins that use small subsets of arginines to recognize RNA targets. (**A**) Global view of the co-crystal structure of FMRP-RGG in complex with G-quadraplex RNA (PDB 5dea) (51). The RGG motif assumes a β-hairpin conformation that contains four arginines. Here and elsewhere, the dashed box surrounding each RNA-protein interface is depicted in **Fig. 7**. (**B**) Global view of the co-crystal structure of the Csy4 endoribonuclease in complex with crRNA (PDB 4al5) (52). The RNA binding domain is an α-helical motif that harbors six arginines. (**C**) Global view of the lowest energy structure of the HIV-1 Tat-TAR ensemble (PDB 6mce) (19). The Tat-ARM contains nine arginines and adopts a linear conformation. (**D**) Global view of the lowest energy structure of the HIV-1 Tat-7SK ensemble (PDB 6mcf) (19). The Tat-ARM contains nine arginines, but unlike the Tat-TAR conformation, it adopts a β-hairpin structure. These global views highlight the structural plasticity of arginine-rich motifs (ARMs) and their tendency to use a subset of arginines for RNA recognition.

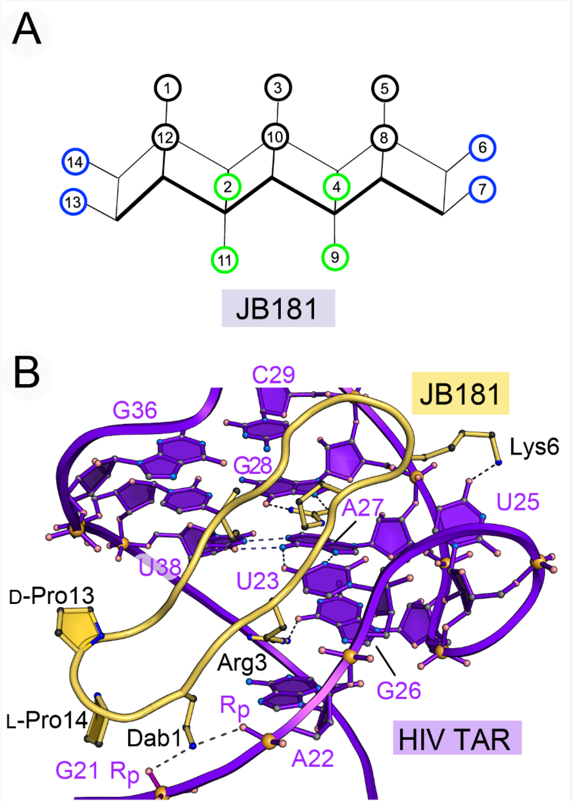

Figure S7 | The ultra-high affinity TAR binding peptide JB181 uses two arginines to make base-specific interactions. (**A**) Schematic drawing of TAR-binding cyclic-peptide JB181 derived from structure-based design (25). The 14-mer adopts an antiparallel β-hairpin conformation with side chains optimized to stabilize the up-down β-strand residues that compose the core fold. Sidechains in black interact with RNA, those in green are exposed to solvent, and blue residues support cyclization. (**B**) Close-up view of JB181 interactions with TAR. JB181 contains four arginines total, wherein Arg3 and Arg5 make base-specific contacts with Gua26 and Gua28. These interactions are similar to Arg47 and Arg49 in the TBPs, where Arg47 forms an arginine-fork interaction (**Fig. S1**). Dab1 (2,4-diaminobutyric acid) makes phosphate backbone interactions with Gua21 and Ade22, similar to Arg48 and Arg52 in TBP6.7 Q48R/T50R (**Fig. 4**).
