# Supplementary material for: Co-crystal structures of HIV TAR RNA bound to lab-evolved proteins show key roles for arginine relevant to the design of cyclic peptide TAR inhibitors
Source: J Biol Chem. 2021 Jan 13;295(49):16470–86. doi: 10.1074/jbc.RA120.015444 (PMC7864049; doi:10.1074/jbc.RA120.015444)
Supplement: Supplementary file 2 — Sai Shashank Chavali [file mmc2.pdf]

Sai Shashank Chavali

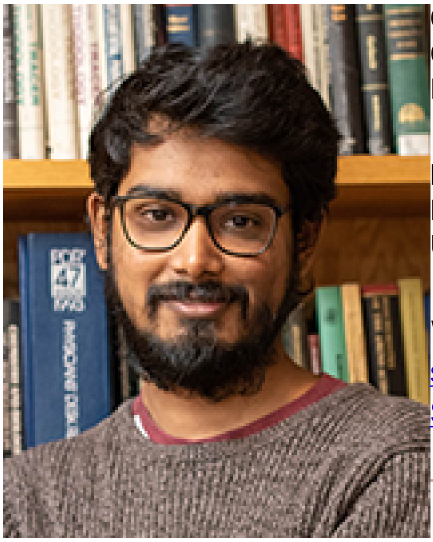

Current position: Ph.D. Student, Biophysics, Structural and Computational Program, University of Rochester School of Medicine and Dentistry, Rochester, New York, USA

Education: M.S., Biophysics, 2019, University of Rochester, Rochester, New York, USA; M.S., Biology, 2016, Rutgers University, Rutgers, New Jersey, USA

Where to find me: https://www.linkedin.com/in/shashankchavali/; https://www.researchgate.net/profile/Shashank_Chavali

---

How did you become interested in this topic?

I was intrigued by structure-based drug discovery as an undergraduate. My first research experience entailed docking small molecules into enzymes with the goal of repurposing drugs. This curiosity prompted me to pursue a master's degree at Rutgers, where I worked to understand the dynamics of membrane proteins using molecular dynamics simulations. I became interested in RNA biophysics when I matriculated to the University of Rochester and joined Prof. Wedekind's lab to conduct my doctoral work. My experiences have made me realize that RNA can be a drug target and that this is an emerging field with great potential. To establish a solid foundation for drug discovery, I have focused my efforts on elucidating the principles of RNA molecular recognition using lab-evolved proteins as a model system. This work is paving the way to design small cyclic peptides that target HIV-1 TAR RNA.

Can you describe an exciting moment you experienced while doing this research?

It was really exciting to see our cyclic peptide derived from a lab-evolved protein retain HIV-1 TAR binding and block HIV-1 Tat's arginine-rich motif from binding. This has positive implications in suppressing HIV transcription.

If you could go back in time and re-do this project, what advice would you give your past self?

I would tell myself to look at experimental results without any bias. I learned that if well-controlled experiments don't show an expected outcome, then there is something else going on that may add greater value to the outcome.

What do you hope to do next?

Going further, I wish to delve into cryo-EM and cryo-ET. Cryo-EM is transforming structural biology and drug discovery. I am currently looking for postdoctoral positions in labs that utilize cryo-EM and cryo-ET techniques to gain an atomic-level understanding of molecular assemblies in disease-relevant molecules.

Where do you seek scientific inspiration?

I find inspiration in structural biology mainly from my advisor Prof. Wedekind and my colleagues. In addition, Richard Feynman's quote, "Everything that living things do can be understood in terms of jiggling and wiggling of atoms," always drives me to think of biological problems at an atomic level.

Read Chavali's article on page 16470.
